# Symbiotic phosphate transporter dynamics in rice expose functional plasticity of the arbuscules

**DOI:** 10.1038/s41467-026-71496-8

**Published:** 2026-04-22

**Authors:** Jennifer McGaley, Martina Orvošová, Ben Schneider, Chai Hao Chiu, Ronelle Roth, Sarah Bowden, Matthew S. Hope, Jayne L. Davis, Warda Khalif, Emma J. Wallington, Uta Paszkowski

**Affiliations:** 1 Crop Science Centre, Department of Plant Sciences, University of Cambridge, Cambridge, United Kingdom; 2 Department of Biochemistry and Biophysics, Stockholm University, Stockholm, Sweden; 3 Department of Biology, University of Oxford, Oxford, United Kingdom; 4 Niab, Cambridge, United Kingdom

**Keywords:** Arbuscular mycorrhiza, Cellular imaging, Plant transporters, Fungal biology, Agriculture

## Abstract

Mutualism in the symbiosis between arbuscular mycorrhizal fungi and plants is based upon the exchange of carbon for soil minerals, with phosphate being of central importance. The exchange of nutrients occurs when the fungus transiently colonises root cells, producing hyphal structures called arbuscules. The movement of phosphate from fungus to plant is well established, however its coordination and regulation at the ephemeral arbuscules remains elusive. Here, non-invasive imaging captures the complete growth and collapse of the arbuscules in unprecedented resolution, revealing heterogeneity in arbuscule development. Tracking the dynamics of rice PHosphate Transporter 1;11 (OsPHT1;11/ PT11) as a proxy for symbiotic phosphate transport shows consistent localisation across diverse arbuscules. However, we uncover phosphate-responsive variability in PT11 abundance, representing an essential, cellular-level layer of nutrient regulation. Such plasticity in arbuscule phosphate uptake capacity evidences uncoupling of arbuscule presence and arbuscule function, thereby demonstrating that arbuscules are not identical units of nutrient exchange.

## Abstract (web summary)

This study uncovers the previously-hidden dynamics of nutrient exchange structures central to the symbiosis between plants and arbuscular mycorrhizal fungi, revealing highly variable development, lifespans and phosphate transport capacities.

## Introduction

Phosphate is one of the most scarcely available yet greatly demanded minerals for plant growth. A phosphate-acquisition solution present in around 80% of plant species, in both natural and agricultural ecosystems across all continents, is the symbiotic association with arbuscular mycorrhizal (AM) fungi^1^. The soil-dwelling AM fungi form extensive networks of hyphae that can collect phosphate alongside other mineral nutrients from beyond the reach of plant roots^2^. The acquired minerals are supplied to the plant in exchange for organic carbon, on which the fungus is entirely dependent^3^. The mycorrhizal-route can contribute up to 100% of a plant’s total phosphorus demand, making it one of the most important elements in plant-fungal trade^4^.

Once foraged by the AM fungal mycelium, phosphate is trafficked to hyphae inside the plant roots, ultimately to specialised, branched exchange structures within plant root cortical cells: the arbuscules^5,6^. Here, phosphate is exported from the fungus before being transported across the enveloping plant-derived, peri-arbuscular membrane (PAM) and into the plant cytosol^7^. This final stage is mediated by specific members of the PHosphate Transporter 1 (PHT1) proton symporter family that are predominantly induced in arbusculated cells^8–15^. The importance of PHT1-mediated phosphate transport in AM symbiosis is demonstrated by mutant studies, in which absence of the respective AM-specific PHT1 protein leads to small, prematurely-collapsing arbuscules, reduced mycorrhizal colonisation, and a diminished mycorrhizal growth response^10,14–17^.

But while the fundamental transfer of phosphate from AM fungi to plants has been well established for over half a century at the level of the whole organism^18^, the cellular-level dynamics remain elusive. Using immuno-localisation or live imaging fluorescent reporters in excised and sectioned roots, symbiotic PHT1 proteins have been found to localise in the PAM surrounding arbuscule fine branches, earmarking this as the domain of phosphate uptake^9,13,19,20^. The techniques employed gave great spatial resolution, but their destructive nature limited the insights to static snapshots. And the arbuscules are far from static: time-course and time-lapse experiments have revealed the transient nature of the arbuscules, with asynchronous growth and collapse over a matter of days^21–23^. The structures are also morphologically diverse, lying on a spectrum between coil and arbuscule, dictated by both plant and fungal partner identity^24^. The result is an ever-changing mosaic of nutrient exchange structures and developmental stages thereof, situated in expanding and contracting colonisation zones within a root system. How symbiotic phosphate uptake is coordinated and regulated at such a heterogeneous and fluid exchange interface remains largely unknown.

In this study, we investigated the spatiotemporal dynamics of the AM-specific phosphate uptake transporter of rice, OsPHT1;11 (PT11 from here on)^15^, as an indicator of when and where phosphate transfer can take place at the arbuscules. By employing high-resolution, live, confocal microscopy alongside a non-invasive, time-lapse imaging technique, we captured the growth and collapse of the arbuscules in unprecedented spatial and temporal resolution, uncovering previously unseen variation in developmental trajectories as well as lifespan. Using a suite of transcriptional and translational fluorescent reporter lines, we mapped out consistent coordination of PT11 at this dynamic interface. But this experimental approach also uncovered a cellular-level layer of nutrient regulation of the symbiotic phosphate importer critical to symbiotic success. The observed capacity for symbiotic nutrient exchange to be fine-tuned at the arbuscule level exposes a distinction between arbuscule presence and function. Coupled with the diversity in development and lifespan, these findings highlight individuality amongst the arbuscules. This has important implications for our methods of assessing AM symbiosis, and raises questions about the abiotic and biotic factors controlling arbuscule form and function.

## Results

### Diversity in arbuscule lifespan and developmental trajectories

To first visualise the spatiotemporal dynamics of arbuscule development and collapse, we sought a ‘neutral’ peri-arbuscular membrane (PAM) marker with no role in arbuscule development or AM colonisation. We employed a fluorescent reporter of the rice AM-specific Secretory Carrier Membrane Protein (SCAMP), *pSCAMP:eGFP-SCAMP*, which had previously been shown to localise to the PAM throughout the arbuscule lifespan, with characteristic distributions at each developmental stage^22,25^. We validated the suitability of this reporter by assessing the AM phenotype of a Tos17 retrotransposon insertion mutant *scamp* allele^22^. At both early and late stages of the symbiosis with *Rhizophagus irregularis, scamp* mutant plants showed no significant difference in colonisation to wild-type plants (Supplemental Fig. S1).

**Fig. 1:**
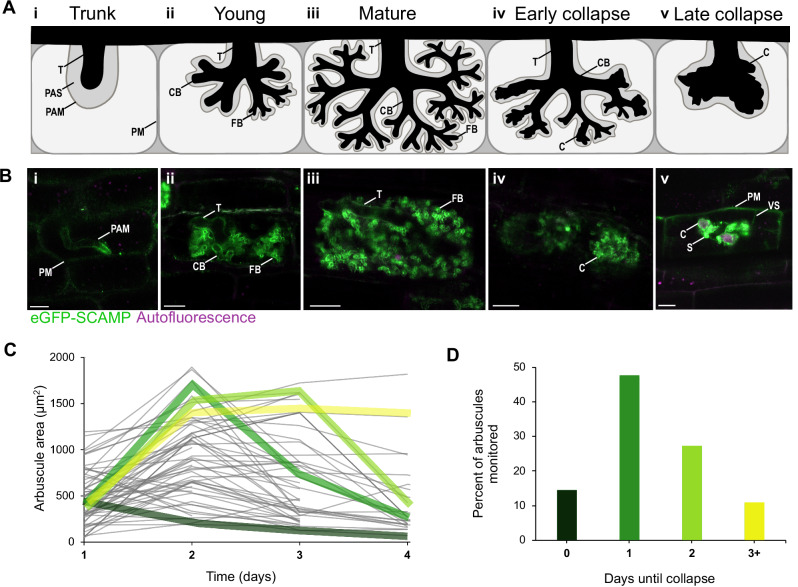
Spatiotemporal dynamics of the arbuscules. **A** Illustration of the distinct developmental stages of the arbuscule. Black depicts arbuscular mycorrhizal fungus, light grey depicts plant cells, and dark grey depicts the apoplast. **B** Representative micrographs are shown of arbuscule developmental stages imaged in live *pSCAMP:eGFP-SCAMP-*expressing rice roots colonised by *R. irregularis* (independent arbuscules). Images are representative of observations made in 3 + independent experiments. Images are maximum intensity z projections. Green = eGFP, magenta = autofluorescence, scale bars = 10 μm. Annotations mark arbuscule trunks (T), coarse branches (CB), fine branches (FB), collapsed branches (C), peri-arbuscular membrane (PAM), peri-arbuscular space (PAS), plasma membrane (PM), SCAMP spots (S) and trans-vacuolar strands (VS). **C** Arbuscule growth trajectories over time. Grey lines indicate the area of individual arbuscules at 24 hr intervals, measured in *pSCAMP:eGFP-SCAMP-*expressing rice plants colonised by *R. irregularis* in AMSlides. Coloured lines highlight the main growth trajectory patterns: immediate collapse (dark green), 1 day until collapse (green), 2 days until collapse (light green), more than 3 days until collapse (yellow). *n* = 56 arbuscules from 5 plants. **D** Quantification of the proportion of arbuscules monitored that showed the developmental trajectories highlighted in (**C**). Raw data for (**C**,** D**) are available in the Source Data file.

High-resolution confocal microscopy of *pSCAMP:eGFP-SCAMP* rice plants colonised by *R. irregularis* was used to define discrete developmental stages and sub-cellular domains of the arbuscule: (i) Trunk, consisting of first unbranched intracellular hypha, in which eGFP-SCAMP localised to the plasma membrane (PM) and PAM (strongest accumulation at trunk tip); (ii) Young arbuscule, consisting of coarse branches plus initial fine branches, with eGFP-SCAMP present in the entire PAM (strongest accumulation at branch tips); (iii) Mature arbuscule, marked by extensive fine branching, filling the cell, with eGFP-SCAMP in the PAM surrounding trunk and branches (strongest signal at fine branch tips); (iv) Early collapse arbuscule, evidenced by clumped branches retracted from the cell boundary, with eGFP-SCAMP localised to trunk and clumped branches; and (v) Late collapse arbuscule, consisting of a single mass of autofluorescent material surrounded by eGFP-SCAMP, with additional eGFP-SCAMP present in the PM, transvacuolar strands and SCAMP ‘spots’ (Fig. 1A, B)^22,25^. eGFP-SCAMP signal was also present in the PM of cells in the vicinity of arbusculated cells (Fig. 1Bi, v).

To understand the temporal dynamics of the arbuscule life-span, we grew *pSCAMP:eGFP-SCAMP* plants in the AMSlide: a custom chamber for non-invasive, live imaging of root interactions^23^. By imaging regions of colonisation every 24 h over multiple days, we tracked individual arbuscules from appearance to collapse and quantified arbuscule life-span (Fig. 1C). While an average of 75% of arbuscules developed and collapsed within a 1- or 2-day period, 15% of arbuscules collapsed in less than 1 day and 10% persisted for more than 3 days (Fig. 1D). Examining arbuscule morphologies at a higher temporal resolution of 2-hour intervals gave further insight (Supplemental Movie S1). While some arbuscules with a 1-day lifespan reached a mature, fine-branched and cell-filling state before collapsing, others collapsed prematurely (Supplementary Movie S1 and Supplementary Fig. S2A–H). This demonstrates the high variability not only in arbuscule lifespan, but also developmental trajectory.

### *PT11* promoter activity is coordinated with arbuscule fine branching

**Fig. 2:**
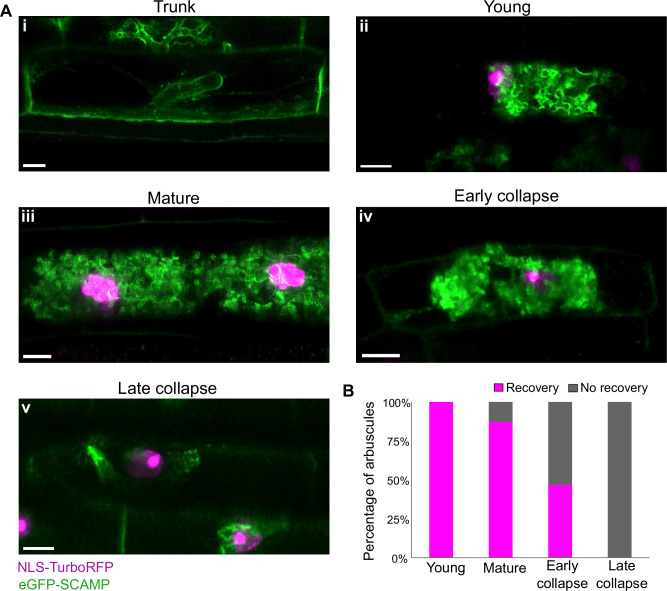
PT11 promoter activity at different arbuscule developmental stages. Rice co-expressing *pPT11:NLS-TurboRFP* and *pSCAMP:eGFP-SCAMP * was imaged live at 6 weeks post inoculation with *R. irregularis*. **A** Representative images are shown of cells hosting (i) arbuscule trunk, (ii) young arbuscule, (iii) mature arbuscule, (iv) early collapsing arbuscule, (v) and late collapsing arbuscule. Images are representative of observations made in 3 independent experiments. Images are maximum intensity z projections. Green = eGFP, magenta = TurboRFP, scale bars = 10 μm. **B** Fluorescence recovery after photobleaching (FRAP) results for rice line co-expressing *pSCAMP:eGFP-SCAMP; pPT11:NLS-TurboRFP* colonised by *R. irregularis* in an AMSlide. Percentage of arbuscules in which the photobleached TurboRFP signal recovered after 4 hours is shown for each arbuscule developmental stage (assigned using eGFP-SCAMP marker distribution, Fig. 1B). *n* = 55 arbuscules from 3 plants. Raw data for (**B**) are available in the Source Data file.

To map the promoter activity of the *PT11* gene onto this temporally dynamic interface, a transcriptional reporter line (*pPT11:NLS-TurboRFP*) was generated and genetically crossed with the *pSCAMP:eGFP-SCAMP* reporter line. Upon colonisation by *R. irregularis*, no TurboRFP signal was detected in cells hosting trunks, identified using eGFP-SCAMP distribution (Fig. 2Ai). Cells with finely-branched arbuscules however, showed intense TurboRFP fluorescence (Fig. 2Aii-iii). TurboRFP was still detected in cells hosting collapsed arbuscules (Fig. 2Aiv-v), however it is known that NLS-tagged fluorophores can remain stable in the nucleus beyond the timeframe of promoter activity^26,27^. A fluorescence recovery after photobleaching (FRAP) assay was therefore performed on *pSCAMP:eGFP-SCAMP; pPT11:NLS-TurboRFP* co-expression plants colonised by *R. irregularis* in AMSlides. While the TurboRFP signal recovered in 88% of cells hosting mature arbuscules, this dropped to 47% at early collapse stages, and 0% at late collapse, showing TurboRFP was no longer being produced (Fig. 2B). *PT11* promoter activity is therefore restricted to stages of arbuscule fine-branching, with decline upon arbuscule collapse.

### PT11 consistently localises to the fine branch domain of the PAM

To investigate how the timing of *PT11* promoter activity corresponds to protein localisation, we employed translational fusion reporters: *pPT11:PT11-eGFP* from Kobae and Hata^19^ and newly-generated *pPT11:PT11-mRFP1*. The *pPT11:PT11-eGFP* line had previously been used to probe PT11 localisation, showing it situated at the arbuscule branches^19^, however, functionality of the fluorescent reporter construct was not checked, nor were the fine-scale dynamics investigated over arbuscule lifetime. To test the functionality of both reporter constructs, a mutant complementation experiment was performed. CRISPR-Cas9 was used to generate two new *pt11* mutant alleles: *pt11-3* (1697 bp deletion spanning promoter and first exon) and *pt11-4* (7 bp deletion in 5’ UTR and 1 bp insertion in first exon) (Supplemental Fig. S3A). At an early stage of colonisation by *R. irregularis*, total colonisation of rice roots of both mutant alleles was lower than wild-type plants (Supplemental Fig. S3B). By a later stage, both alleles showed a severe, significant reduction in total colonisation level compared to wild-type, with 7- and 14-fold reduction in arbuscule presence and 47- and 38-fold reduction in vesicle presence in *pt11-3* and *pt11-4*, respectively (Supplementary Fig. S3B). Closer inspection of colonisation in *pt11-3* revealed tiny, clumped arbuscules, in contrast to the cell-filling, finely-branched arbuscules in wild-type plants (Supplementary Fig. S4A). Arbuscule size: plant cell size ratio measurements showed a significant shift towards smaller arbuscule size classes in *pt11-3* (Supplementary Fig. S4B), and morphological examination confirmed this was caused by an over-representation of collapse-stage arbuscules (Supplementary Fig. S4C).

Crossing *pPT11:PT11-eGFP* or *pPT11:PT11-mRFP1* into the *pt11-3* mutant background fully restored total AM colonisation levels and the abundance of all colonisation structures when inoculated with *R. irregularis* (Supplementary Fig. S5). As a negative control, the transcriptional reporter *pPT11:NLS-TurboRFP* was also crossed into *pt11-3* and showed no significant difference in colonisation to *pt11-3* (Supplementary Fig. S5A). This assured the functionality of the translational reporter lines, validating subsequent experimental observations.

To ensure fluorophore choice did not impact the observed protein localisation^28^ the *pPT11:PT11-eGFP* and *pPT11:PT11-mRFP1* lines were crossed to generate a co-expression line. Similar reporter distributions and fluorescence intensities were seen in the PAM regardless of fluorophore tag (Supplementary Fig. S6). Upon removal from the PAM, mRFP1 could be detected in the vacuole (unlike eGFP), likely due to this protein’s tolerance to lower pH^29^. To investigate any fluorophore-induced mislocalisation or fluorophore cleavage, the distribution of fluorescent signal from PT11-eGFP and PT11-mRFP1 reporters were compared to the distributions of ‘free’ eGFP and mRFP1 proteins in arbusculated cells (Supplemental Fig. S7). While the ‘free’ fluorescent proteins appeared as a nucleocytoplasmic haze throughout the cell, the PT11-fused fluorescent proteins clearly outlined arbuscule branches (Supplementary Fig. S7). Together, these data validate the use of both translational reporters to map PT11 dynamics.

**Fig. 3:**
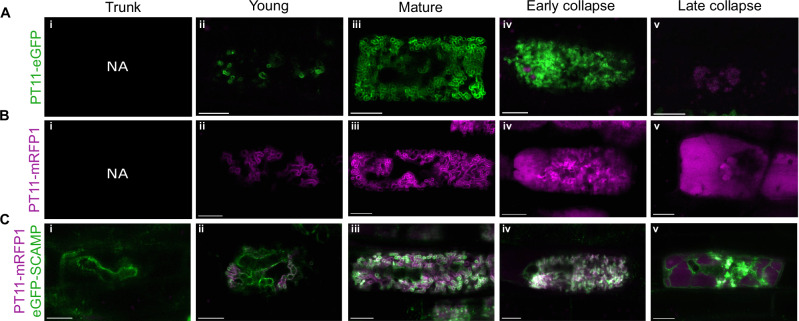
Localisation of fluorescently-tagged PT11 at different arbuscule developmental stages in rice. Rice plants were imaged live at 6 weeks post inoculation with *R. irregularis*. Representative micrographs are shown of (i) trunk, (ii) young, (iii) mature, (iv) early collapse and (v) late collapse arbuscules in each rice reporter line. Images are representative of observations made in 3 independent experiments. **A** Rice expressing *pPT11:PT11-eGFP*. Green = eGFP, magenta = autofluorescence. **B** Rice expressing *pPT11:PT11-mRFP1*. Magenta = mRFP1, green = autofluorescence. **C** *pSCAMP:eGFP-SCAMP; pPT11:PT11-mRFP1* co-expression line. Green = eGFP, magenta = mRFP1 (separate channels, overlays, and fluorescence intensity transects shown in Supplementary Fig. S8). Micrographs are maximum intensity z projections. NA denotes no observable fluorescent signal at that arbuscule developmental stage. Scale bars = 10 μm.

High resolution, live, confocal microscopy of *pPT11:PT11-eGFP, pPT11:PT11-mRFP1*, and a *pSCAMP:eGFP-SCAMP; pPT11:PT11-mRFP1* co-expression line allowed characterisation of PT11 localisation throughout arbuscule lifespan. In the *pPT11:PT11-eGFP* and *pPT11:PT11-mRFP1* lines, no cells hosting trunk-stage arbuscules could be detected (Fig. 3Ai, 3Bi). The absence of PT11 at this stage was confirmed by co-expression alongside *pSCAMP:eGFP-SCAMP*. Here, the trunks were clearly highlighted by eGFP-SCAMP, but PT11-mRFP1 was undetectable (Fig. 3Ci and Supplementary Fig. S8i). In cells hosting young arbuscules, PT11 was absent from the PAM subdomains around trunks and coarse branches but localised to the tips of the first fine branches (Fig. 3Aii, Bii, Cii, Supplementary Fig. 8ii). At mature arbuscules, PT11 showed a more even distribution on the PAM around the entire fine branches, again mostly undetectable around trunks and coarse branches (Fig. 3Aiii, Biii, Ciii and Supplementary Fig. S8iii). This localisation pattern persisted in cells hosting early-stage collapsing arbuscules, with PT11 still present on clumped fine-branches, but PT11 was absent from the PAM at collapsed arbuscules, with only stable vacuolar mRFP1 visible in the *pPT11:PT11-mRFP1* line (Fig. 3Bv, Cv and Supplementary Fig. S8v).

To investigate how representative these snapshot images were of PT11 dynamics over the course of a single arbuscule’s lifespan, time-lapse imaging was performed on the *pSCAMP:eGFP-SCAMP; pPT11:PT11-mRFP1* co-expression line. Imaging at 2-hour intervals captured the appearance of PT11 on the PAM upon fine-branching, and removal upon collapse (Supplementary Movie S2). This was independent of arbuscule developmental trajectory or lifespan: PT11 was observed only at young and mature-stage arbuscules around the fine branches regardless of whether the arbuscule collapsed after < 12 h or > 50 h (Supplementary Movies S3–S6, Supplementary Fig. S9), and whether it reached maturity before collapsing or collapsed prematurely (Supplementary Movies S3–S6).

### PT11 dynamics are consistent across arbuscules of diverse AM fungi

**Fig. 4:**
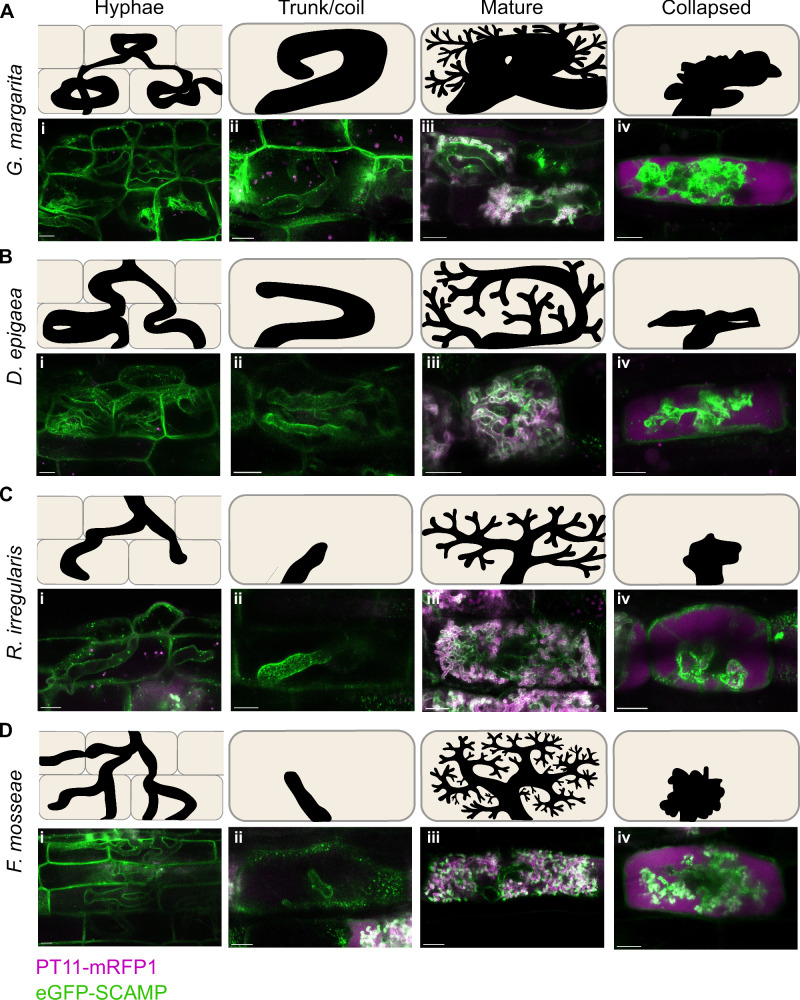
Localisation of PT11-mRFP1 and eGFP-SCAMP at symbiotic structures of rice roots colonised by diverse arbuscular mycorrhizal fungi. **A**–**D** Representative live images of (i) intracellular hyphae, (ii) arbuscule trunks/coils, (iii) mature arbuscules, and (iv) late collapsed arbuscules in *pSCAMP:eGFP-SCAMP; pPT11:PT11-mRFP1* expressing rice roots at 6 weeks post inoculation with (**A**) *Gigaspora margarita*, (**B**) *Diversispora epigaea*, (**C**) *Rhizophagus irregularis* or (**D**) *Funneliformis mosseae*. Images are representative of observations made in 2 independent experiments. Micrographs are maximum intensity z projections. Magenta = mRFP1, green = eGFP, scale bars = 10 μm.

To explore the consistency of PT11 dynamics, the *pSCAMP:eGFP-SCAMP; pPT11:PT11-mRFP1* co-expression line was inoculated with taxonomically diverse AM fungal species: *Gigaspora margarita* (Gigasporaceae, Diversisporales), *Diversispora epigaea (*Diversisporaceae, Diversisporales), *Funneliformis mosseae* (Glomeraceae, Glomerales) or *Rhizophagus irregularis* (Glomeraceae, Glomerales)^30^. All species from each of the three families successfully colonised rice (Supplementary Fig. S10). The fungi developed morphologically diverse symbiotic structures, which were imaged live using the *pSCAMP:eGFP-SCAMP; pPT11:PT11-mRFP1* line (Fig. 4). The intracellular hyphae of both *G. margarita* and *D. epigaea* in outer cell layers of the root formed extensive, swollen coils (Fig. 4Ai, Bi). Once in the cortex, *G. margarita* developed further thick coils, from which fine branches emerged to produce arbuscules (Fig. 4Ai-ii). *D. epigaea* arbuscules also showed a coil-like trunk, but the arbuscule branches were much coarser than *G. margarita* (Fig. 4Bi-ii). *F. mosseae* formed a more branched hyphal network in outer root cell layers, while *R. irregularis* hyphae were less prone to coiling or branching (Fig. 4Ci, Di). In the cortex, both *R. irregularis* and *F. mosseae* developed straight trunks, which dichotomously branched to form arbuscules (Fig. 4Cii-iii, Dii-iii). The branches of *F. mosseae* arbuscules were extremely fine, while *R. irregularis* arbuscule branches were of intermediate thickness between *D. epigaea* and *F. mosseae* (Fig. 4Ciii, Diii). Collapse-stage arbuscules of all four AM fungal species looked similar, namely a central clump of plant-fungal material (Fig. 4Aiv, Biv, Civ, Div).

Regardless of fungal species, phylogenetic group, or the morphology of symbiotic structures, PT11 was visible only in cells hosting branched arbuscules (Fig. 4). While outlined by eGFP-SCAMP, no PT11-mRFP1 was detected around intracellular hyphae in outer root cell layers, coils in cortical cells, or arbuscule trunks in cortical cells. In the arbusculated cells, PT11-mRFP1 was still absent from the trunks and most abundant on the finest branches, whether this was < 1 µm diameter *F. mosseae* branches or > 1.5 µm diameter *D. epigaea* branches (Fig. 4Biii, 4Diii). Upon collapse, mRFP1 could only be detected in the vacuole, implying that PT11 expression ceased at this stage. These data suggest that coordination of PT11 is consistent in symbioses with diverse AM fungi.

### PT11 abundance on the PAM is variable

**Fig. 5:**
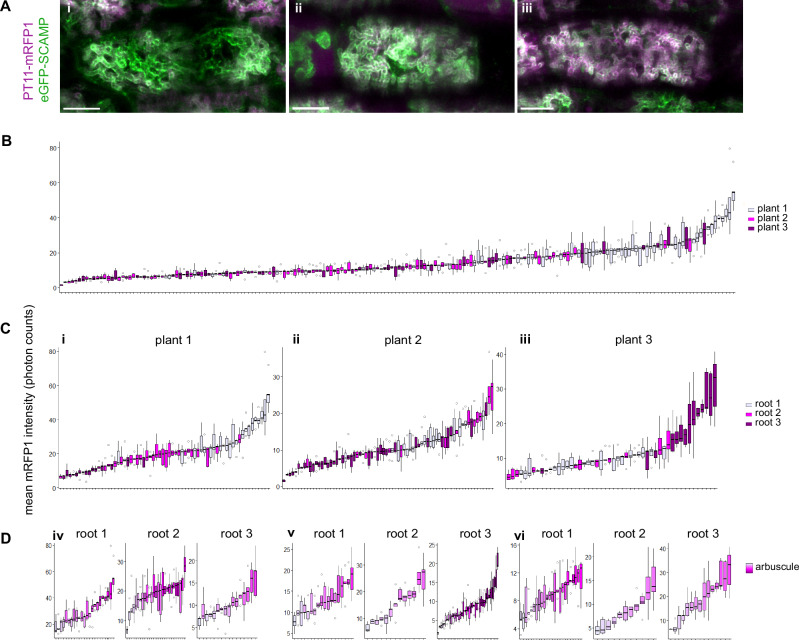
Variation in PT11-mRFP1 protein abundance at mature arbuscules in rice colonised by *R. irregularis*. Live imaging of *pSCAMP:eGFP-SCAMP; pPT11:PT11-mRFP1 -*expressing rice plants was performed at 6 weeks post inoculation. **A** Example micrographs of arbuscules with (i) low, (ii) medium and (iii) high mRFP1:eGFP mean fluorescence intensity ratio. Images are maximum intensity z projections. Green = eGFP, magenta = mRFP1, scale bars = 10 μm. **B**–**D** Mean mRFP1 fluorescence intensity measured from five branch tips per arbuscule, all arbuscules of one colonisation zone per root, three roots per plant, three plant replicates (*n* = 66, 72 and 50 arbuscules for plants 1, 2 and 3, respectively). Data is ordered by median fluorescence intensity per arbuscule and displayed (**B**) ungrouped, where colour depicts plant replicates, (**C**) grouped by plant, where colour depicts root replicates, and (**D**) grouped by root, where arbuscules are coloured by median intensity relative to arbuscules within the same root. Plots show median and interquartile range (coloured box), data range excluding outliers (whiskers) and outliers (open circles, defined as datapoints outside 1.5* interquartile range). Raw data for (**B**–**D**) are available in the Source Data file.

Live imaging the *pSCAMP:eGFP-SCAMP; pPT11:PT11-mRFP1* co-expression reporter revealed unexpected qualitative differences in the relative abundance of PT11 on the PAM of morphologically and developmentally analogous arbuscules (Fig. 5A). To better understand at which organisational level this variation exists, mean mRFP1 fluorescence at branch tips was measured in all mature arbuscules across multiple colonisation zones, large lateral roots, and plant individuals. Restricted maximum likelihood analysis of mixed effect models showed that the greatest degree of variance occurred at the inter-root level (52% of total) (Fig. 5C), closely followed by inter-arbuscule (44%) (Fig. 5D). Inter-plant variance was only responsible for 4% of the total (Fig. 5B). To ensure that the observed inter-root and inter-cell variability was not the result of variation in imaging depth, the same analysis was performed on mRFP1:eGFP ratio (Supplementary Fig. S11). This showed similarly high variability in relative protein abundances between cells and roots, confirming the high variability of PT11 abundance on the PAM at mature arbuscules.

### PT11 abundance on the PAM is responsive to plant nutrient demands

**Fig. 6:**
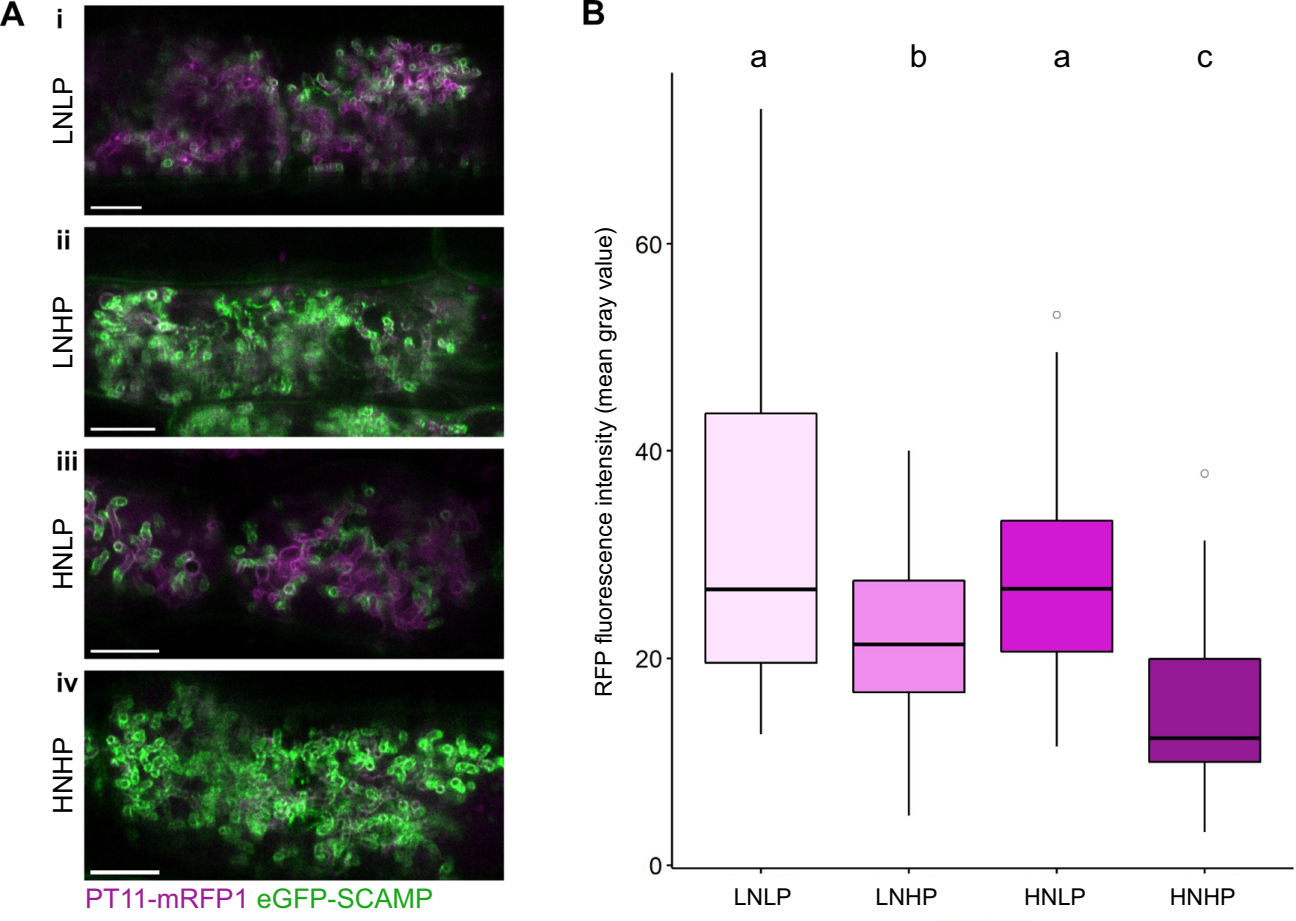
Variable abundance of PT11-mRFP1 and eGFP-SCAMP at the arbuscules in rice plants grown under different nutrient fertilisation regimes. Plants were imaged live at 6 weeks post inoculation with *R. irregularis*, after 4 weeks of fertilisation with low nitrogen and low phosphate (LNLP), low nitrogen and high phosphate (LNHP), high nitrogen and low phosphate (HNLP), or high nitrogen and high phosphate (HNHP). **A** Representative micrographs of arbuscules in rice plants co-expressing *pPT11:PT11-mRFP1* and *pSCAMP:eGFP-SCAMP*, grown under each nutrient regime. Images are maximum intensity z projections. Green = eGFP-SCAMP, magenta = mRFP1, scale bars = 10 μm. **B** Quantification of mean mRFP1 fluorescence at all mature arbuscules of one colonisation zone per root, three roots per plant, three plants per nutrient regime (*n* = 34 (LNLP), 37 (LNHP), 39 (HNLP) and 33 (HNHP) arbuscules). Letters depict result of Kruskall Wallis test (*p* = 7.35 × 10^−9^) with Post-Hoc Dunn testing (*p* < *0.05*). Plots show median and interquartile range (coloured box), data range excluding outliers (whiskers) and outliers (open circles, defined as datapoints outside 1.5* interquartile range). Raw data for (**B**) are available in the Source Data file.

We hypothesised that the variable abundance of PT11 in individual arbusculated cells may be the result of differences in local nutrient conditions. To investigate this, *pSCAMP:eGFP-SCAMP*; *pPT11:PT11-mRFP1* co-expression lines were inoculated with *R. irregularis* and grown under different nutrient regimes: low nitrate (LN, 0.05 mM) or high nitrate (HN, 3 mM) and either low phosphate (LP, 0.025 mM) or high phosphate (HP, 0.25 mM) fertilisation. As expected, the total colonisation level was suppressed by high-phosphate fertilisation, with ~60% root length colonisation by arbuscules in HNLP, but only ~15% in HNHP (Supplementary Fig. S12). Colonisation levels were intermediate in LNHP and LNLP (Supplementary Fig. S12). Live imaging revealed consistent localisations of PT11-mRFP1 and eGFP-SCAMP on the PAM enveloping the arbuscules, as previously described (Fig. 6A). However, arbuscules in LP-grown plants were visibly more PT11-mRFP1 dominated, while arbuscules in HP-grown plants were more eGFP-SCAMP dominated (Fig. 6Ai-iv). Quantification of PT11-mRFP1 fluorescence confirmed that plants grown under LP hosted arbuscules with significantly higher mRFP1 fluorescence intensity than those grown under HP (Fig. 6B), proving that PT11 abundance on the PAM is responsive to plant phosphate demand.

**Fig. 7:**
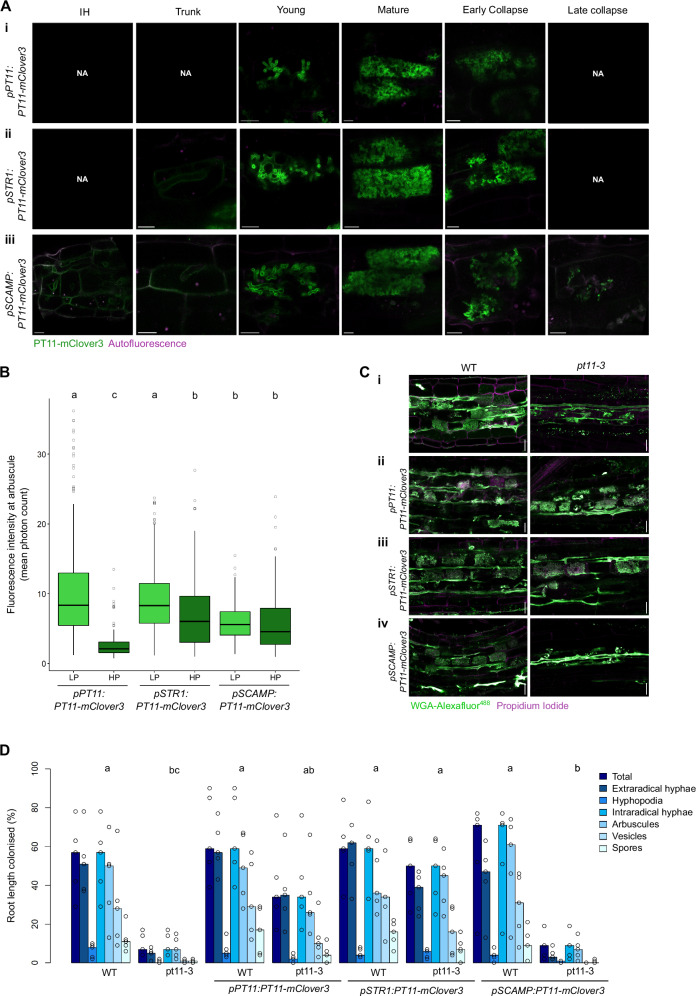
Localisation, nutrient responsiveness and arbuscular mycorrhizal colonisation phenotypes of PT11 promoter-swap reporter rice lines assessed at 6 weeks post inoculation with *R.* *irregularis*. **A** Representative micrographs are shown of PT11-mClover3 localisation in cells hosting intraradical hyphae (IH), trunk, young, mature, early collapse or late collapse arbuscules when expressed under the (i) native *PT11* promoter, (ii) *STR1* promoter, or (iii) *SCAMP* promoter. Images are representative of observations made in 2 independent experiments. Roots were imaged live. Green = eGFP, magenta = autofluorescence. Micrographs are maximum intensity z projections. NA denotes no observable fluorescent signal at that arbuscule developmental stage. Scale bars = 10 μm. **B** Mean fluorescence intensity of mClover3 at arbuscules in rice expressing *pPT11:PT11-mClover3, pSTR1:PT11-mClover3*, or *pSCAMP:PT11-mClover3* when grown under low phosphate (LP, 25 μM) or high phosphate (HP, 250 μM) fertilisation for 4 weeks. Letters depict the result of Kruskall Wallis test (*p* = 2.2 × 10^−16^) with Post-Hoc Dunn testing (*p* < *0.05*). All mature arbuscules in one colonisation zone per root, three roots per plant, three plants per genotype and treatment were imaged (*n* = 311/122 arbuscules (*pPT11:PT11-mClover3)*, 336/140 arbuscules (*pSTR1:PT11-mClover3*), and 304/151 arbuscules (*pSCAMP:PT11-mClover3*) for LP and HP treatments, respectively). Plots show median and interquartile range (coloured boxplot), data range excluding outliers (whiskers) and outliers (open circles, defined as datapoints outside 1.5* interquartile range). **C** Representative images of arbuscular mycorrhizal colonisation in wild-type (WT) or *pt11-3* mutant rice roots (i) without a reporter construct, or expressing (ii) *pPT11:PT11-mClover3*, (iii) *pSTR1:PT11-mClover3*, or (iv) *pSCAMP:PT11-mClover3* under low phosphate fertilisation. Images are maximum intensity z projections. Green = WGA-Alexafluor^488^, magenta = propidium iodide, scale bars = 10 μm. **D** Root length colonisation quantification for promoter-swap constructs in wild-type (WT) or *pt11-3* backgrounds under low phosphate fertilisation. Graph shows percent of root hosting extraradical hyphae, hyphopodia, intraradical hyphae, arbuscules, vesicles, spores and any intraradical structure (Total). Letters depict result of Kruskall Wallis test (*p* = 1.36 × 10^−3^*, n = *five plants per genotype) with Post-Hoc Dunn testing (*p* < *0.05*). Raw data for (**B**, **D**) are available in the Source Data file.

Due to stark qualitative differences in mRFP1 and eGFP intensity at arbuscules of plants grown under HP versus LP, eGFP-SCAMP intensity alone was also examined. Surprisingly, the opposite pattern to PT11-mRFP1 was seen, with higher eGFP-SCAMP abundance on the PAM of HP-grown plants (Supplemental Fig. S13). This implies the differences in PT11-mRFP1 abundance are indeed reflective of symbiotic phosphate transporter regulation, not just a universal feature of PAM-resident proteins. *pPT11:PT11-mClover3* reporter lines showed the same trend as *pPT11:PT11-mRFP1* when grown under HP versus LP, verifying that properties of the fluorescent tag were not responsible for the observed fluorescence intensity differences under different nutrient regimes (Fig. 7B).

### Precise regulation of PT11 is essential for AM symbiosis

Having uncovered precise spatiotemporal dynamics and arbuscule-level regulation of PT11, we next investigated whether these are necessary for functional AM symbiosis. A set of promoter-swap constructs were generated to alter PT11 dynamics and/or nutrient regulation. Subsequently, the ability of these promoter-swap constructs to rescue the *pt11-3* mutant phenotype was tested. The promoter-swap constructs consisted of PT11 C-terminally tagged with mClover3, driven by the promoter of the arbuscule-specific nutrient transporter *STR1* (*pSTR1:PT11-mClover3)*, the promoter of *SCAMP* (*pSCAMP:PT11-mClover3*), or the native *PT11* promoter as a control (*pPT11:PT11-mClover3)*. The *STR1* promoter was selected because it is an arbuscule-specific nutrient transporter and was hence anticipated to share dynamics and/or regulatory patterns with PT11^31,32^. The *SCAMP* promoter was selected due to SCAMP’s broader localisation domain and opposite phosphate-regulation pattern to PT11 (Fig. 1B and Supplementary Fig. S13).

To examine any changes to PT11 localisation, arbuscules in the roots of each promoter-swap reporter line (in the wild-type background) colonised by *R. irregularis* were imaged live. When driven by the native promoter, PT11 showed the anticipated fine-branch PAM-localisation at young and mature arbuscules (Fig. 7Ai). When driven by the *STR1* promoter, PT11 remained restricted to arbusculated cells, but was now present in the PAM surrounding the trunks and coarse branches in addition to the native fine-branch localisation (Fig. 7Aii). The *SCAMP* promoter produced a very similar distribution to *pSTR1:PT11-mClover3* in arbusculated cells, with additional presence in the endoplasmic reticulum of cells hosting intracellular hyphae, and as aggregates around collapsed arbuscules (Fig. 7Aiii).

To test if these promoters altered the nutrient-regulatability of PT11, each promoter-swap reporter line (in the wild-type background) was inoculated with *R. irregularis* and grown under high phosphate (HP, 0.25 mM) or low phosphate (LP, 0.025 mM). Total AM colonisation of all reporter lines was suppressed under HP (Supplementary Fig. S14). Mirroring previous experiments, PT11-mClover3 abundance at the PAM was significantly reduced under HP when driven by the *PT11* promoter (Fig. 7B). The same was observed when PT11-mClover3 was expressed under the *STR1* promoter (Fig. 7B). However, when driven by the *SCAMP* promoter, nutrient responsiveness was lost, with no significant difference in mClover3 fluorescence intensities at the arbuscules between LP and HP treatments (Fig. 7B).

This selection of constructs therefore allowed us to dissect the relative importance of PT11 localisation versus nutrient regulation, as it included lines showing native localisation and phosphate-regulation (*pPT11:PT11-mClover3)*, expanded localisation but correct phosphate-regulation (*pSTR1:PT11-mClover3)* and expanded localisation and abolished phosphate-regulation (*pSCAMP:PT11-mClover3)*. To test the functionality of the constructs, each line was crossed with the *pt11-3* mutant. Sibling *pt11-3* or wild-type F3 plants containing the reporter constructs were then grown with *R. irregularis*. As previously described, *pt11-3* roots hosted smaller, clumped arbuscules (Fig. 7Ci) and significantly lower colonisation levels compared to wild-type (Fig. 7D). While the *pPT11:PT11-mClover3* and *pSTR1:PT11-mClover3* constructs could rescue arbuscule morphology in the *pt11-3* mutant background (Fig. 7Cii-iii) and restore colonisation to wild-type levels (Fig. 7D), this was not observed for *pSCAMP:PT11-mClover3*. Colonisation level and arbuscule morphology in *pt11-3*; *pSCAMP:PT11-mClover3* plants was indistinguishable from *pt11-3* (Fig. 7Civ, Fig. 7D). Therefore, only the constructs that maintained arbuscule-level nutrient regulation of PT11, not PT11 localisation, could retain PT11 function and facilitate successful AM colonisation.

## Discussion

The dynamics of the arbuscules have long been enigmatic. While high-resolution imaging has previously revealed their intricate structures, the temporal aspect has been elusive^33^. And while pioneering work by Kobae and Fujiwara^22^ measured arbuscule lifespans, resolution limitations prevented observation of their morphology. Here, we achieved both: high-resolution imaging of the arbuscules over their entire lifespans from ‘birth to death’.

Monitoring the arbuscules over time exposed inter-arbuscule variation in developmental trajectory, adding another layer of complexity to the arbuscules, which were already known to have diverse morphologies and lifespans^22–24^. This raises the question of what the decisive cues are to never fully develop, or to mature and persist for many days. Symbiotic phosphate transport may be involved, as premature arbuscule collapse is seen in mutants of arbuscule-specific PHT1 transporters^14–16,34^. Supporting this, Kobae et al.^25^ reported that high phosphate application transiently impairs new arbuscule development, proposing that the plant favours direct phosphate uptake mechanisms over the symbiotic route. While the same study suggested that phosphate application did not impact the lifespan of mature arbuscules, it followed arbuscules for only 22 h after a single phosphate-application^25^. Further time-lapse imaging of arbuscules under different nutrient conditions may help get to the bottom of the variable, and on the whole surprisingly short, arbuscule life-span^35,36^. In addition, the non-invasive, observational approaches developed in this work open the door to understanding the dynamics of many other arbuscule-intrinsic processes, such as symbiotic signalling and cellular-reorganisation, as well as the relationship between intraradical and extraradical hyphal dynamics^37^.

Investigating the coordination of symbiotic phosphate importers during this highly dynamic arbuscule lifespan gave a very consistent picture. Regardless of fungal partner, arbuscule morphology, developmental trajectory or lifespan, PT11 was expressed at ‘young’ and ‘mature’ stages, localising evenly around the finest branches (Figs. 3, 4). The absence of PT11 from coils of *G. margarita* aligns with observations of *Gigaspora rosea* by Kobae and Hata ^19^ and reinforces the arbuscule fine branches as the sites of symbiotic nutrient exchange. Restriction of phosphate transporters to the arbuscule fine branches has previously been observed in rice colonised by *Funneliformis mosseae* (formerly *Glomus mosseae*)^19^ and *Paraglomus occultum*^38^*, Medicago truncatula* colonised by *Diversispora epigaea* (formerly *Glomus versiforme*)^9,20^ and *Glycine max* colonised by *R. irregularis*^13^. This may reflect the most efficient nutrient transport domain, as is the case for many fractally-organised biological structures^39^, or correspond to the location of maximal phosphate release by AM fungi, who generally exhibit tip-oriented growth and secretion^7,40^. Future work to investigate whether this localisation is common to other plant symbiotic nutrient transporters, and how they compare to fungal equivalents on the other side of the peri-arbuscular space would shed light on this.

Despite consistent localisation of PT11, extensive live imaging revealed highly variable PT11 abundance between arbuscules (Fig. 5), suggesting morphologically analogous arbuscules can have markedly different phosphate uptake capacities, indicative of local, possibly cell-autonomous, regulation. Inter-arbuscule variation in protein abundance has not been previously reported, the closest to our knowledge being the nonsynchronous promoter activity of *StPT3* in arbusculated cells of potato, however arbuscule developmental stage was not examined^41^. There are reports of some AM structures being entirely metabolically inactive, however ‘presence’ and ‘vitality’ have not been assessed for the same colonised regions^36,42^. Cellular-level differences remain indetectable by bulk root gene expression analyses, and even with fluorescent reporters, only become apparent in co-expression lines. We therefore suspect that this inter-arbuscule variability is a widespread phenomenon. Recent breakthroughs in applying single-cell and spatial transcriptomics to AM symbiosis should help reveal the extent of inter-arbuscule variability in other plant and fungal species, and for other symbiotic genes^43–45^. Combinatorial approaches will also be required to ascertain how differences in phosphate transporter abundance correlate with actual phosphate transport at the single-arbuscule scale.

It could not initially be concluded if the difference in PT11 abundance was a consequence of gene expression ‘noise’^46^, or a symbiosis-relevant cellular-level of phosphate-uptake regulation. We therefore turned to a factor already known to regulate AM symbiosis: plant nutrient status. Fertilisation with HP not only supressed the total number of arbuscules, as is well established^47^ (Supplementary Fig. S12), but also the PT11 abundance at the PAM, potentially indicating reduced phosphate uptake capacity of the remaining arbuscules (Fig. 6). This extra layer of regulation at the nutrient transporter level has not been reported before, as bulk changes in gene expression cannot be uncoupled from concomitant changes in colonisation level and arbuscule development^48^. The HP-suppression of PT11 abundance was dampened under LN, implying a regulatory role of nitrogen as well as phosphorus status (Fig. 6). This has been previously observed at the level of total colonisation and arbuscule morphology^49,50^. Our findings add a ‘fine-tuning’ element, whereby colonisation levels and arbuscule morphology can be identical between different nutrient conditions (e.g., HNLP and LNHP), but actual nutrient transporters can reflect the true mineral demands of the plant. Such uncoupling of arbuscule presence and arbuscule function means that arbuscules cannot be considered identical ‘units of nutrient exchange’, and may be one explanatory factor for the frequently observed disparity between colonisation level and nutrient exchange or mycorrhizal growth response^51^.

Despite the systemic shifts in average PT11 abundance under different fertilisation regimes, there was still great inter-arbuscule variation, implying additional local regulation (Fig. 6). This may reflect variable phosphate supply from the fungal partner^52^, or differences in cellular phosphate concentrations caused by a cell’s colonisation history or colonisation status of neighbouring cells. Cell-level regulation of subsequent symbiotic phosphate uptake may be key to maintaining cellular phosphate homoeostasis^53^. Recent work has identified arbusculated cell-expressed members of the phosphate-sensing SPX family, and revealed their importance in AM colonisation^54–56^. These sensors may be the means by which arbusculated cell phosphate levels are detected and integrated into the regulation of further symbiotic phosphate uptake.

Having observed that symbiotic phosphate transport is spatiotemporally precisely coordinated and regulated at the arbuscule level, we wished to investigate the relevance of this for AM symbiosis. The *SCAMP* promoter was selected to expand the localisation of PT11, which it did in arbusculated cells, but in cells hosting intraradical hyphae PT11 appeared in the ER (Fig. 7A). This is consistent with observations by both Pumplin et al.^57^ and Kobae et al.^19^, where ectopically-expressed symbiotic phosphate transporters are ER-retained. Further, using the *SCAMP* promoter abolished the phosphate-responsiveness of PT11 abundance, in contrast to the pattern of *pSCAMP:eGFP-SCAMP* (more abundant under HP) or *pPT11:PT11-mClover3* (less abundant under HP). This suggests that while PT11 expression timing and localisation is largely transcriptionally controlled (Fig. 2), in agreement with Pumplin et al.^57^, abundance of PT11 involves post-translational mechanisms, of which many have been described for PHT1-family transporters^58^.

The *STR1* promoter was selected to retain the native nutrient regulation of PT11, which it did, however, it additionally expanded the domain of PT11 localisation (Fig. 7A, B). This serendipitous finding allowed unravelling of the relative importances of PT11 localisation versus nutrient regulation: only the constructs maintaining proper nutrient responsiveness of PT11, not necessarily localisation, could rescue the *pt11-3* mutant phenotype (Fig. 7C, D). It should be noted that domain of PT11 localisation was more expanded in *pSCAMP:PT11-mClover3* than *pSTR1:PT11-mClover3*. However, the lack of dominant-negative effect of *pSCAMP:PT11-mClover3* in the wild-type background led us to negate this as the cause of failed *pt11-3* complementation (Fig. 7C, D and Supplementary Fig. S14). Instead, we propose that the restriction of PT11 to the PAM around the arbuscule fine branches represents the most efficient location for symbiotic phosphate uptake, not an essential feature of AM symbiosis. The nutrient responsiveness of PT11 on the other hand appears to be essential for AM colonisation.

Overall, this work sheds light on the extreme dynamism and diversity of the arbuscules. The finding that arbuscules not only have variable morphologies and lifespans, but also differing development and function, may reflect the tuneability required for such an intimate symbiosis between decentralised partner organisms. The arbuscule-level of complexity offers a mechanism by which plant and fungal demands and offerings can be balanced more precisely and rapidly than broader changes to the symbiosis, such as alterations to colonisation level or engagement with new symbiotic partners. It also allows systemic needs to be integrated with local cellular conditions across heterogeneous root and hyphal networks. However, this ‘arbuscule individuality’ has important implications for our interpretation of AM colonisation: the presence of an arbuscule (e.g., in a stained root sample) does not guarantee its functionality or longevity. Going forward, it will be key to determine how arbuscule-level dynamics translate into overall fungus-plant nutrient transfer and symbiotic outcome.

## Methods

### Mutant rice lines

The *scamp* mutant line in *Oryza sativa* spp. Japonica cv. Nipponbare was generated by Miyao et al.^59^ and obtained from Yoshihiro Kobae^22^. The *scamp* mutant allele contains a Tos17 retrotransposon insertion in the third exon (Supplementary Fig. S1A). Plants were genotyped using the primers listed in Supplementary Table S3.

To generate *PT11* mutants in *Oryza sativa* spp. Japonica cv. Nipponbare by CRISPR-Cas9, the Csy-type (CRISPR system yersinia) ribonuclease 4 (Csy4) ribozyme approach, part of the toolkit described in Čermák et al.^60^, was used. For each gene, two small guide RNAs (sgRNAs) were designed using CRISPR-P^61^. sgRNAs with the highest on-score and lowest off-score with minimal and most dissimilar off-targets were chosen. Primers containing the necessary restriction sites were designed using the webtool of Čermák et al.^60^ (discontinued) to amplify Csy4 spacers flanked by sgRNAs via PCR. PCR products were digested, ligated and assembled into pMOD_B2112 with a GoldenGate^62^ reaction involving SapI, AarI and T7 ligase (New England Biolabs). pMOD_B2112 plasmids with sgRNA incorporated were then assembled into pTRANS_250d with another GoldenGate reaction to obtain plasmids ready for *Agrobacterium tumefaciens* mediated plant transformation. The construct sequence was verified via Sanger Sequencing (Source BioScience).

*Oryza sativa* spp. Japonica cv. Nipponbare rice callus was generated by plating surface-sterilised mature seed, with embryo axes removed, on N6DT medium (3.95 g/L N6 basal salts, 30 g/L sucrose, 300 mg/L casein hydrolysate, 100 mg/L myo-inositol, 2878 mg/L proline, 0.5 mg/L nicotinic acid, 0.5 mg/L pyridoxine HCl, 1 mg/L thiamine HCl, 37.3 mg/L Na_2_EDTA, 27.8 mg/L FeSO_4_, 2 mg/L 2,4-D Na salt, 150 mg/L Timentin, 4 g/L Gelrite, pH5.8). Plates were sealed with Parafilm and cultured in the dark at 28 ^o^C for 21 days. Callus was cut into 2–4 mm pieces, plated on fresh N6DT and cultured as before for a further 4 days. Transformation of the rice callus pieces and hygromycin selection was carried out as previously described^63^. *PT11* edits in regenerated plants were assessed by Sanger Sequencing (Source BioScience) of PCR products using the primers in Supplementary Table S1. Subsequent genotyping for *pt11-3* allele was carried out using primers listed in Supplementary Table S3. Homozygous mutant *pt11-3* and *pt11-4* plants lacking the Cas9 construct (genotyping for *HPT*, Supplementary Table S3) were used in experiments.

### Fluorescent reporter rice material

*Oryza sativa* spp. Japonica cv. Nipponbare transformed with *pSCAMP:eGFP-SCAMP:tNos* and *pPT11:PT11-eGFP:tNos* were acquired from Yoshihiro Kobae^19,22^. All other fluorescent expression cassettes were generated in this work by Golden Gate cloning^62,64^ using the construct modules listed in Supplementary Table S2. Intermediate constructs containing the fluorescent reporter cassettes flanked by Gateway attL1 and attL2 sites were verified by Sanger Sequencing (Source BioScience) before recombining into binary vector pEW343-R1R2 and transforming into *Agrobaterium tumefeciens* strain EHA105 as previously described^63^. Constructs were transformed into *Oryza sativa* spp. Japonica cv. Nipponbare, as stated above. Primary transformant plants were screened for fluorescence by confocal laser scanning microscopy on an SP8 or Stellaris 8 FALCON/FLIM microscope (Leica Biosystems) and two independent lines per construct taken forward for seed-setting and further experiments. Subsequent genotyping was carried out using primers listed in Supplementary Table S2.

Genetic crossing was carried out to generate co-expression lines and mutant-complementation lines. Plants of each parent line were grown to the flowering stage. On the morning of flowering, flowers of designated ‘female’ panicles were vacuum de-emasculated with a vacuum pump (DIVAC 1.2 L, Leybold) bagged, and placed in the vicinity of designated ‘males’. At midday, growth chamber (Conviron) temperature was elevated to 32 ^o^C and humidity to 80%, inducing synchronised flowering. Pollen was transferred from designated ‘males’ to prepared ‘females’ by gently tapping panicles together. Pollen transfer was repeated at midday for two subsequent days. F1 seeds were harvested after three weeks and dried at 50 ^o^C for two weeks before germination and genotyping (Supplementary Table S3).

### Genomic DNA extraction and PCR genotyping

2 cm leaf samples were placed in 2 mL microcentrifuge tubes (Eppendorf) with two 4 mm glass balls and frozen at − 80 ^o^C. Tissue was ground in a Genogrinder (Spex Sample Prep), for 15 s bursts at 1400 strokes per minute until homogenised. 300 µL KCl extraction buffer (1 M KCl; 100 mM Tris-HCl; 10 mM EDTA) was added before tubes were vortexed and incubated in a heat block (Thermomixer compact, Eppendorf) at 98 ^o^C for 10 minutes. Supernatant was transferred to 300 µL 100% isopropanol and centrifuged at 15000 x *g* for 15 min (Eppendorf). Pellet was washed by further centrifugation with 70% ethanol for 10 minutes. DNA was dissolved in ultrapure water before use in PCR reaction using GoTaq G2 Polymerase (Promega).

### Fungal strains

For most experiments, plants were inoculated with spores of *Rhizophagus irregularis* (DAOM197198) isolated from axenic dual culture with carrot root organ culture (ROC)^65^. ROC was agitated in citrate buffer (0.01 M; pH6.0) for 1 hour before collecting spores in a 45 µm sieve (VWR). For the diverse AM fungal experiment, crude inoculum of *R. irregularis* (DAOM197198) was used alongside *Gigaspora margarita* (BEG34) and two strains from the Swiss Collection of Arbuscular Mycorrhizal Fungi: *Funneliformis mosseae* (SAF11) or *Diversispora epigaea* (SAF117). Crude inoculum was produced as described by Säle et al.^66^.

### Plant growth

De-husked rice seeds were surface sterilised by agitating in 3% (v/v) hypochlorite solution for 15 min followed by three washes with sterile water. Seeds were plated on 0.6% (w/v) bactoagar and incubated in the dark for 5 days at 32 ^o^C. For colonisation assessment and most imaging experiments, seedlings were planted into 120 mm Conetainers (Ray Leach) containing sterile silica sand and the AM fungal inoculum of choice, either 300 spores of *R. irregularis* spores (DAOM 197198) from carrot root organ culture, or 4% (v/v) crude inoculum. AMSlide3 chambers were set-up with 100 *R. irregularis* spores as previously described^23^.

All plants were grown in controlled environment rooms (Conviron) under 12 h days of 300 uE light, 28 °C, relative humidity 65%, and nights of 20 °C. Plants were watered with reverse-osmosis (RO) water until sand was saturated three times per week for their first week. Subsequently, plants received modified half-strength Hoaglands solution (Supplementary Table S5) with 0.025 mM phosphate and 0.01% (w/v) sequestrene iron supplement (Syngenta) twice per week and RO water once per week. Where not otherwise stated, this fertilisation regime continued until harvest. For nutrient regime experiments, in weeks 3–6 plants received modified half-strength Hoaglands solution amended to contain low phosphate (LP, 0.025 mM) or high phosphate (HP, 0.25 mM), and low nitrate (LN, 0.05 mM) or high nitrate (HN, 3 mM) (recipe in Supplementary Table S6).

### AM fungal staining and quantification

To assess AM colonisation level, roots were harvested, washed, stained with Trypan Blue and colonisation level quantified using a compound brightfield microscope (Olympus) and modified gridline intersect method, as previously described^67^. To assess arbuscule morphology and developmental stage, harvested roots were immersed in 50% (v/v) ethanol overnight before transferring to 20% (w/v) KOH for 3 days. Roots were washed three times with RO water and incubated in 0.1 M HCl for two hours before further two washes with RO water and one with 1 x phosphate buffered saline solution (PBS). Roots were incubated in a 0.3 μg/mL solution of wheat germ agglutinin (WGA) conjugated with Alexafluor^488^ (Invitrogen) in PBS at 4 ^o^C for 2 weeks. Prior to imaging, roots were counterstained with 5 μg/mL propidium iodide (Sigma-Aldrich) in PBS. All arbuscules of one colonisation zone per root, three roots per plant, three plants per genotype were imaged by confocal laser scanning microscopy (SP8 or Stellaris 8 FALCON/FLIM, Leica Microsystems). Images were captured using a 40x water immersion objective, tuneable, pulsed, white-light laser and hybrid photodetectors (HyD, Leica Microsystems) set to the wavelengths listed in Supplementary Table S4. Z-stack micrographs of the relevant arbuscule volume were captured.

### Live-cell confocal microscopy of excised roots

Root systems of fluorescent reporter lines were gently washed to remove sand and screened under a fluorescence stereomicroscope (M205 FA, Leica Microsystems) to identify roots expressing the relevant fluorescent protein. These roots were excised from the plant and immediately mounted whole in RO water on a microscope slide and imaged on a confocal laser scanning microscope using a 40 x water immersion objective, tuneable pulsed, white-light laser and hybrid detectors (SP8 or Stellaris 8 FALCON/FLIM, Leica Microsystems). Laser and detector settings are listed in Supplementary Table S4. Images were taken of a minimum of three roots per plant and three plants per genotype in every experiment. Z-stack micrographs of the relevant root or arbuscule volume were captured.

### Timelapse microscopy

AMSlide3 chambers were assembled and used as previously described^23^. Briefly, rice seedlings and *R. irregularis* were co-cultivated in AMSlides until colonised (10–14 days) before non-invasive live confocal laser scanning microscopy (Stellaris 8 FALCON/FLIM, Leica Biosystems). Images of colonised root regions were taken every 2 or 24 h for 2-5 days, with chambers returned to controlled environment rooms (Conviron) between imaging. As previously published, the progression of colonisation is not affected by repeated imaging in the AMSlide system^23^. Images were captured using a 40 x water immersion objective and laser and detector settings listed in Supplementary Table S4.

### Fluorescence recovery after photobleaching

Rice lines co-expressing *pSCAMP:eGFP-SCAMP* and *pPT11:NLS-TurboRFP* were grown with *R. irregularis* in AMSlide3 chambers as previously described until colonisation was present (10–14 days)^23^. Arbuscules at different developmental stages were identified by the distribution of eGFP-SCAMP. Nuclei were continuously scanned using TurboRFP imaging settings with laser power adjusted to 100% until no TurboRFP signal could be detected. Plants were then returned to controlled environment chambers. After 4 h, the same arbuscules were imaged and scored as ‘recovered’ or ‘not recovered’ (Fig. 2B) depending on whether TurboRFP was once more detectable.

### Image processing and analysis

FIJI/ImageJ was used for all image processing^68^, including channel overlays, maximum intensity projections of arbuscule z-stacks and timelapse image alignment using ‘Align by ROI’. FIJI/ImageJ was also used for the following methods of image analysis.

- Arbuscule size analysis was carried out on micrographs from WGA- and propidium iodide-stained roots. Arbuscule perimeters (taken at largest point in z-stack of ‘WGA-Alexafluor488’ channel) and cell boundaries (using the ‘propidium iodide’ channel) were traced using the polygon selections tool, and the area measured. The arbuscule area: cell area ratio was calculated for each arbusculated cell. A minimum of 30 arbuscules per plant and three plants per genotype were analysed.
- Fluorescence intensity profiles were generated by drawing transects through structures of interest using the Straight Line tool, and producing ‘Profile Plots’ for each channel.
- Reporter fluorescence intensity at the arbuscules was measured by tracing arbuscule perimeters at their largest point in z-stacks using the Freehand Line and mean fluorescence intensity of each channel measured. A minimum of 50 arbuscules from three roots per plant, three plants per genotype and treatment were measured.
- Reporter fluorescence intensity at branch tips was calculated by tracing arbuscule branch tips with the Freehand Line tool with line thickness set to 3 pixels, before measuring mean fluorescence intensity. Five arbuscule branches were measured per arbuscule, minimum of 50 arbuscules per root, three roots per plant, three replicate plants.

Grey values are reported for datasets acquired from SP8 confocal microscope (Leica Microsystems) with HyD detectors in digital mode. Photon counts are reported for data acquired from Stellaris 8 FALCON/FLIM with HyDX and HyDS detectors in photon-counting mode (Leica Microsystems).

### Graphics and statistics

Base R^69^ and the packages ggplot2^70^ and lme4^71^ were used for all graphs and statistical tests. Where datasets were non-parametric, significant differences were tested using the Kruskal-Wallis chi-squared test with post-hoc Dunn test for comparisons between multiple groups. To test significant differences between two normally distributed groups, T-tests were used. Restricted maximum likelihood analysis of linear mixed effect models was carried out using the lmer function of the lme4 package. All statistical tests used are stated in figure legends. Inkscape version 1.0.2 was used to create all illustrations (Figs. 1A, 4A–D)^72^.

### Reporting summary

Further information on research design is available in the Nature Portfolio Reporting Summary linked to this article.

## Supplementary information

Supplementary Information

Description of Additional Supplementary Files

Supplemental Movie 1

Supplemental Movie 2

Supplemental Movie 3

Supplemental Movie 4

Supplemental Movie 5

Supplemental Movie 6

Reporting Summary

Transparent Peer Review file

## Source data

Source Data

## Data Availability

All quantitative data generated in this study are published as source data alongside this paper. Methods used are detailed in full in the methods section or have been previously published^23^. Rice gene names are from the Rice Genome Annotation Project release 7 (https://rice.uga.edu/) as follows: *OsSCAMP* (LOC_Os03g38590), *OsPT11* (LOC_Os01g46860), *OsSTR1* (LOC_Os09g23640)^73^. Source data are provided in this paper.
