# Supplementary material for: Symbiotic phosphate transporter dynamics in rice expose functional plasticity of the arbuscules
Source: Nat Commun. 2026 Apr 22;17:5450. doi: 10.1038/s41467-026-71496-8 (PMC13282384; doi:10.1038/s41467-026-71496-8)
Supplement: Supplementary file 1 — Supplementary Information [file 41467_2026_71496_MOESM1_ESM.pdf]

# Supplementary information for: Symbiotic phosphate transporter dynamics in rice expose functional plasticity of the arbuscules.

Jennifer McGaley[1*], Martina Orvošová[1], Ben Schneider[2], Chai Hao Chiu[1], Ronelle Roth[1,3], Sarah Bowden[4], Matthew S. Hope[4], Jayne L. Davis[4], Warda Khalif[1], Emma J. Wallington[4], Uta Paszkowski[1*]

*Corresponding authors (Jennifer McGaley, jcm99@cam.ac.uk ; Uta Paszkowski, up220@cam.ac.uk)

[1]Crop Science Centre, Department of Plant Sciences, University of Cambridge, Cambridge, CB3 0LE, United Kingdom

[2] Department of Biochemistry and Biophysics, Stockholm University, Stockholm, 104 05, Sweden

[3] Department of Biology, University of Oxford, Oxford, OX1 3RB, United Kingdom

[4] Niab, Cambridge, CB24 9NZ, United Kingdom

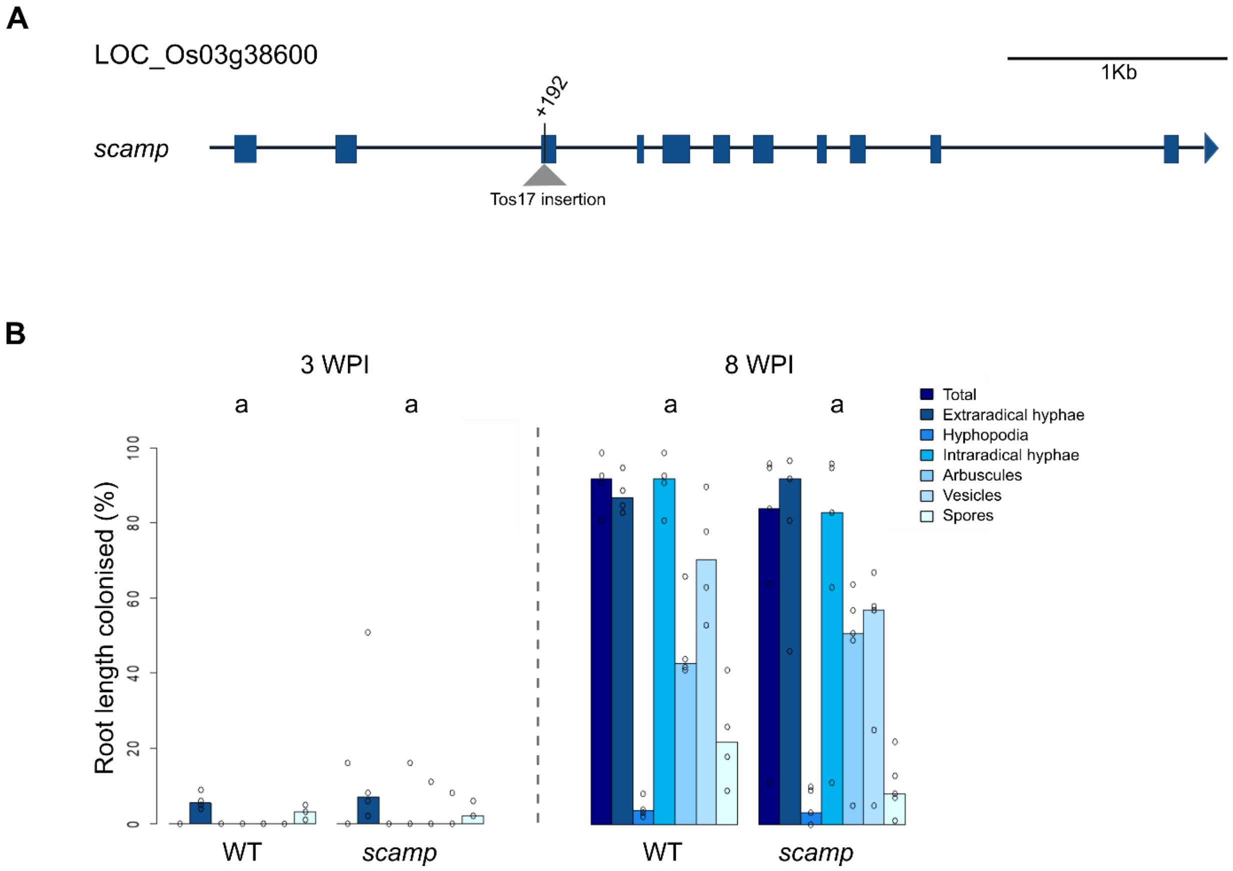


**Figure S1.** Allele structure and arbuscular mycorrhizal colonisation quantification for the rice *scamp* mutant. **A**) Gene structure for rice *SCAMP* gene with annotated location of Tos17 insertion in *scamp* mutant allele. Blue rectangles represent exons. **B**) Root length colonisation quantification for wild-type (WT) and *scamp* mutant rice at 3- and 8-weeks post inoculation with *Rhizophagus irregularis.* Graph shows percent of root hosting extraradical hyphae, hyphopodia, intraradical hyphae, arbuscules, vesicles, spores and any intraradical structure (Total). Letters depict result of Kruskall Wallis test on 'Total' colonisation level with Post-Hoc Dunn test ($p < 0.05$, $n = 4$ plants per genotype and timepoint). Raw data for **B** are available in the Source Data file.

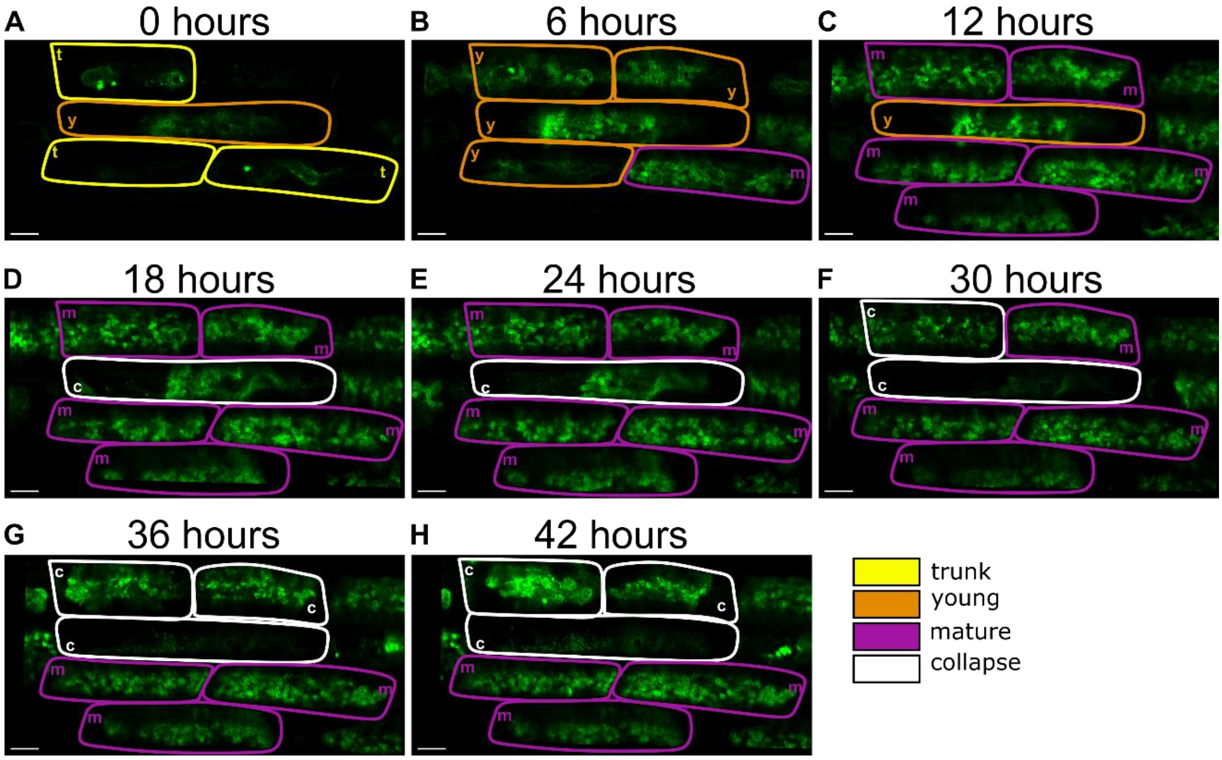


**Figure S2.** Static snapshots of arbuscule developmental trajectories (shown in Supplemental Movie 1). **A-H**) Region of *R. irregularis* colonisation in rice expressing *pSCAMP:eGFP-SCAMP,* grown in an AM Slide and imaged at 2-hour intervals for 42 hours. Images are maximum intensity z projections. Green = eGFP, scale bars = 10 µm. The arbuscule developmental stages, trunk (yellow, t), young (orange, y), mature (magenta, m) and collapse (white, c) are labelled at each timepoint, showing diverse developmental trajectories.

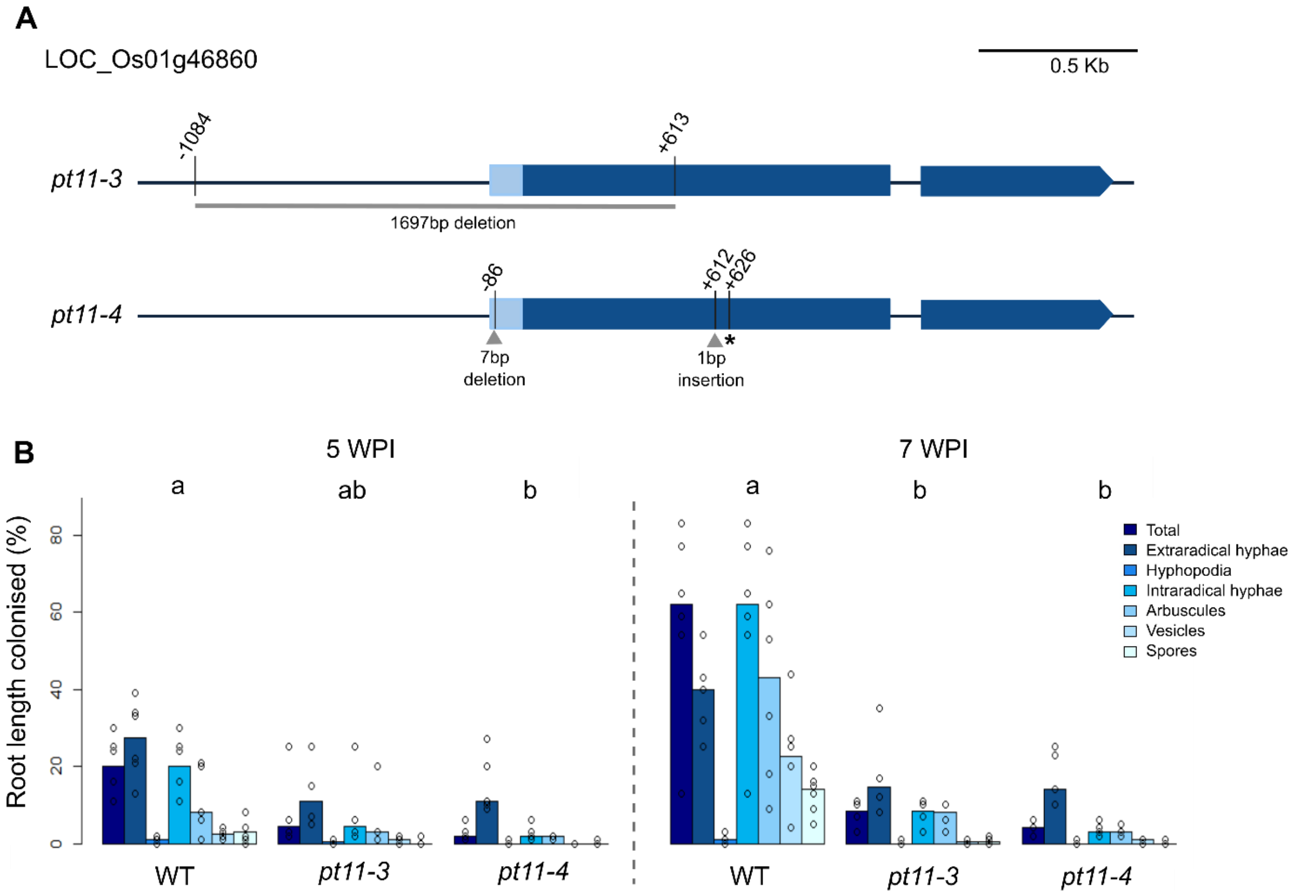


**Figure S3.** Allele structures and arbuscular mycorrhizal colonisation quantification for *pt11-3* and *pt11-4* mutant rice plants. **A)** Gene structure for *PT11* gene with annotated CRISPR edits in each mutant allele. Light blue rectangles represent 5'UTR, dark blue represents exons. Asterisk denotes premature stop codon. **B)** Root length colonisation quantification for wild-type (WT), *pt11-3* and *pt11-4* rice plants at 5 and 7 weeks post inoculation with *R. irregularis.* Graph shows percent of root hosting extraradical hyphae, hyphopodia, intraradical hyphae, arbuscules, vesicles, spores and any intraradical structure (Total). Letters depict result of Kruskall Wallis test with Post-Hoc Dunn test on 'Total' colonisation ($p$< 0.05, $n$= 6 (WT), 4 (*pt11-3*) and 5 (*pt11-4)* plants per timepoint). Raw data for **B** are available in the Source Data file.

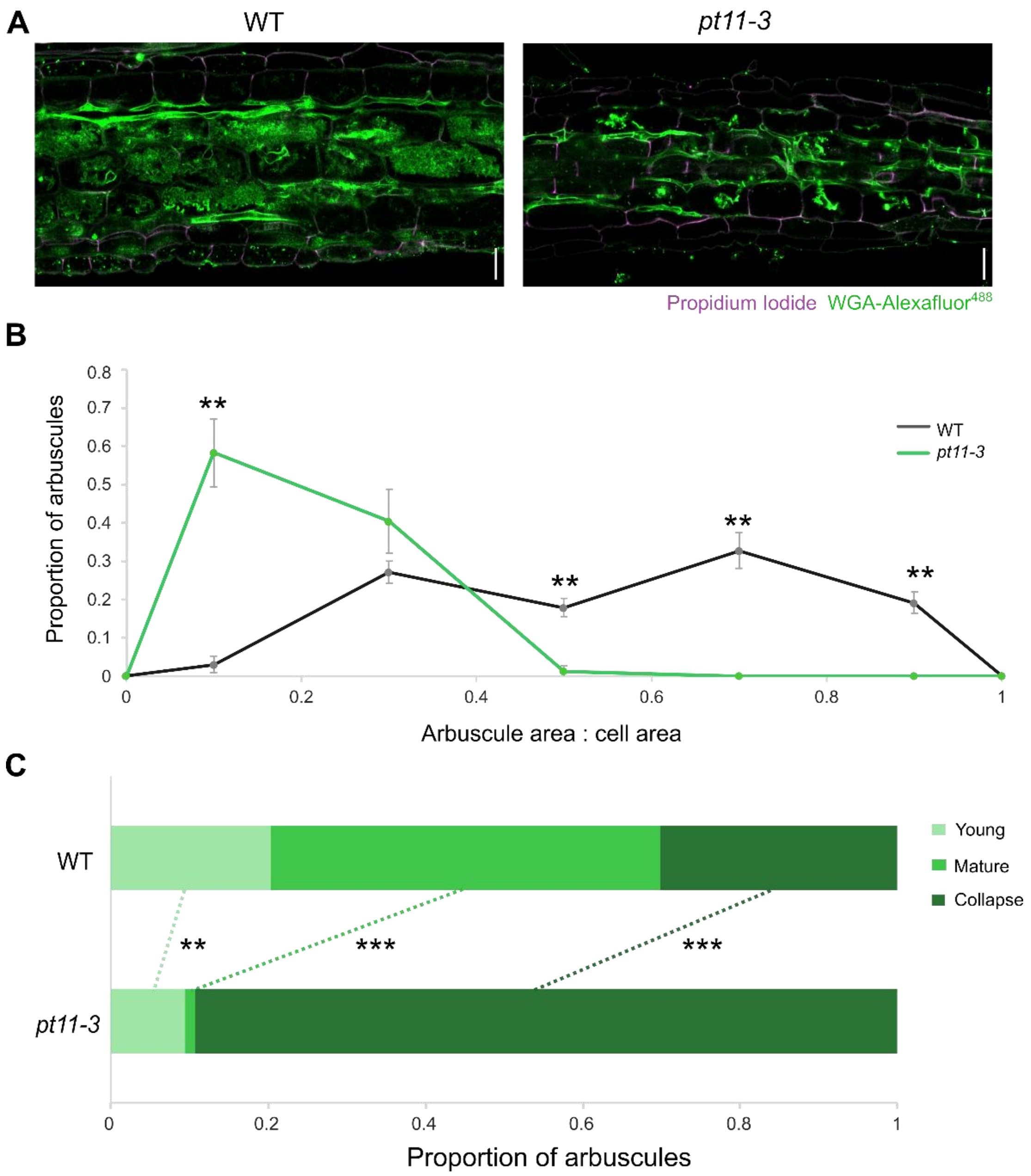


**Figure S4.** Morphology and developmental stage quantification of arbuscules in *pt11-3* mutant rice plants. Plants were assessed at 6 weeks post inoculation with *R. irregularis.* **A**) Representative images of arbuscular mycorrhizal colonisation in wild-type (WT) or *pt11-3* mutant rice roots. Images are maximum intensity z projections. Green = WGA-Alexafluor$^{488}$, magenta = propidium iodide, scale bars = 20 μm. **B**) Size class distributions (measured by arbuscule area:cell area ratio) of arbuscules in wild-type versus *pt11-3*. Minimum of 50 arbuscules measured per plant, three plants per genotype ($n$ = 162 (WT) and 158 (*pt11-3*)). Proportions of arbuscules in each size class were compared between wild-type and *pt11-3* (** = $p < 0.01$, Two-sample T-test). **C**) Proportions of young, mature and collapse-stage arbuscules in *pt11-3* compared to wild-type (** = $p < 0.01$, *** = $p < 0.001$, Two-sample T-test). Raw data for **B-C** are available in the Source Data file.

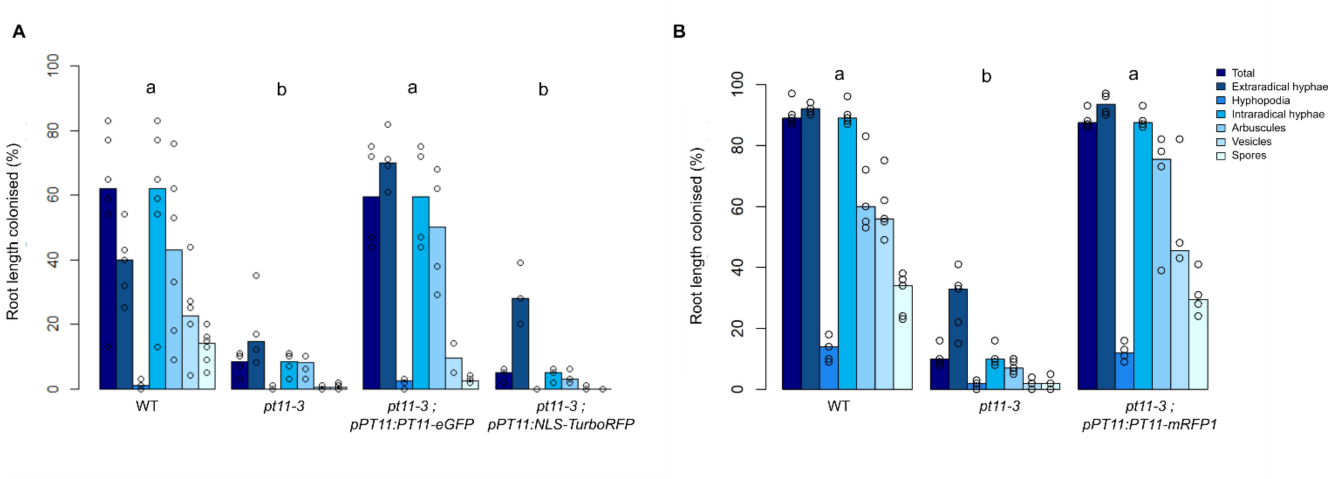


**Figure S5.** Arbuscular mycorrhizal colonisation quantification for PT11 reporter complementation lines. Arbuscular mycorrhizal colonisation was assessed in wild-type (WT) and *pt11-3* alongside *pt11-3* plants expressing (**A**) *pPT11:PT11-eGFP* or *pPT11:NLS-TurboRFP* or (**B**) *pPT11:PT11-mRFP1* at 6 weeks post inoculation with *R. irregularis.* Graph shows percent of root hosting extraradical hyphae, hyphopodia, intraradical hyphae, arbuscules, vesicles, spores and any intraradical structure (Total). **A**) Letters depict result of Kruskall Wallis test on 'Total' colonisation with Post-Hoc Dunn test ($p < 0.05$, $n$= 6 (WT), 4 (*pt11-3*) 4 (*PT11*-eGFP) and 3 (*NLS-TurboRFP*) plants). **B**) Letters depict result of Kruskall Wallis on 'Total' colonisation with Post-Hoc Dunn test ($p < 0.05$, $n$= 5 (WT), 5 (*pt11-3*) 4 (*PT11*-mRFP) plants). Raw data are available in the Source Data file.

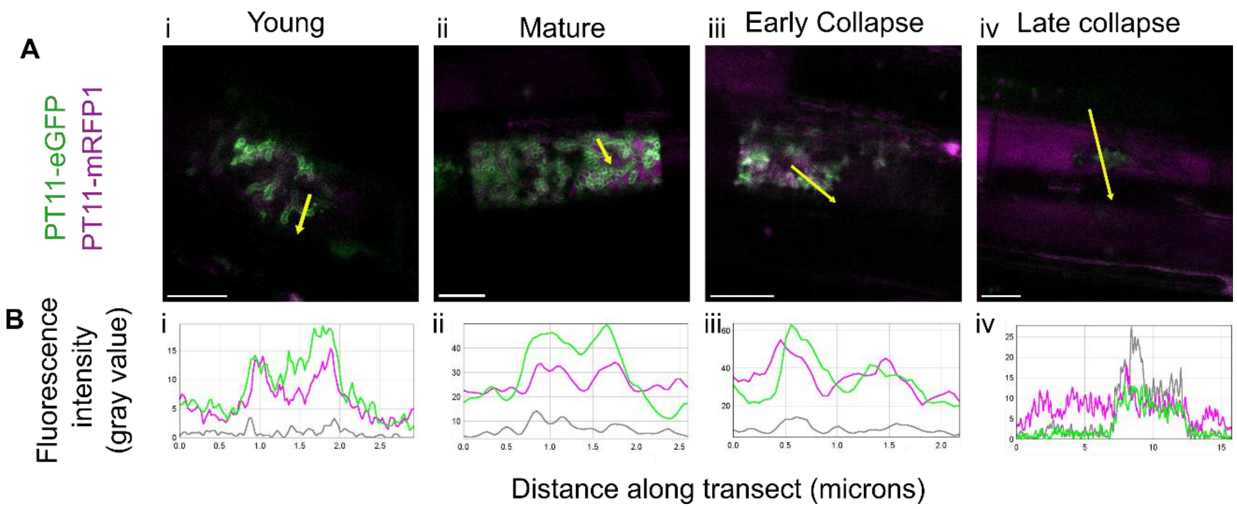


**Figure S6.** Localisation of GFP- and mRFP1-tagged PT11 at different arbuscule developmental stages. Rice plants co-expressing *pPT11:PT11-eGFP* and *pPT11:PT11-mRFP1* were live imaged at 6 weeks post inoculation with *R. irregularis.* **A**) Representative micrographs are shown of (i) young, (ii) mature, (iii) early collapse, and (iv) late collapse arbuscules. Images are single optical slices. Images are representative of observations made in 2 independent experiments. Green = eGFP, magenta = mRFP1, scale bars = 10 µm. **B**) eGFP (green) and mRFP1 (magenta) fluorescence intensities along corresponding micrograph transect in **A**.

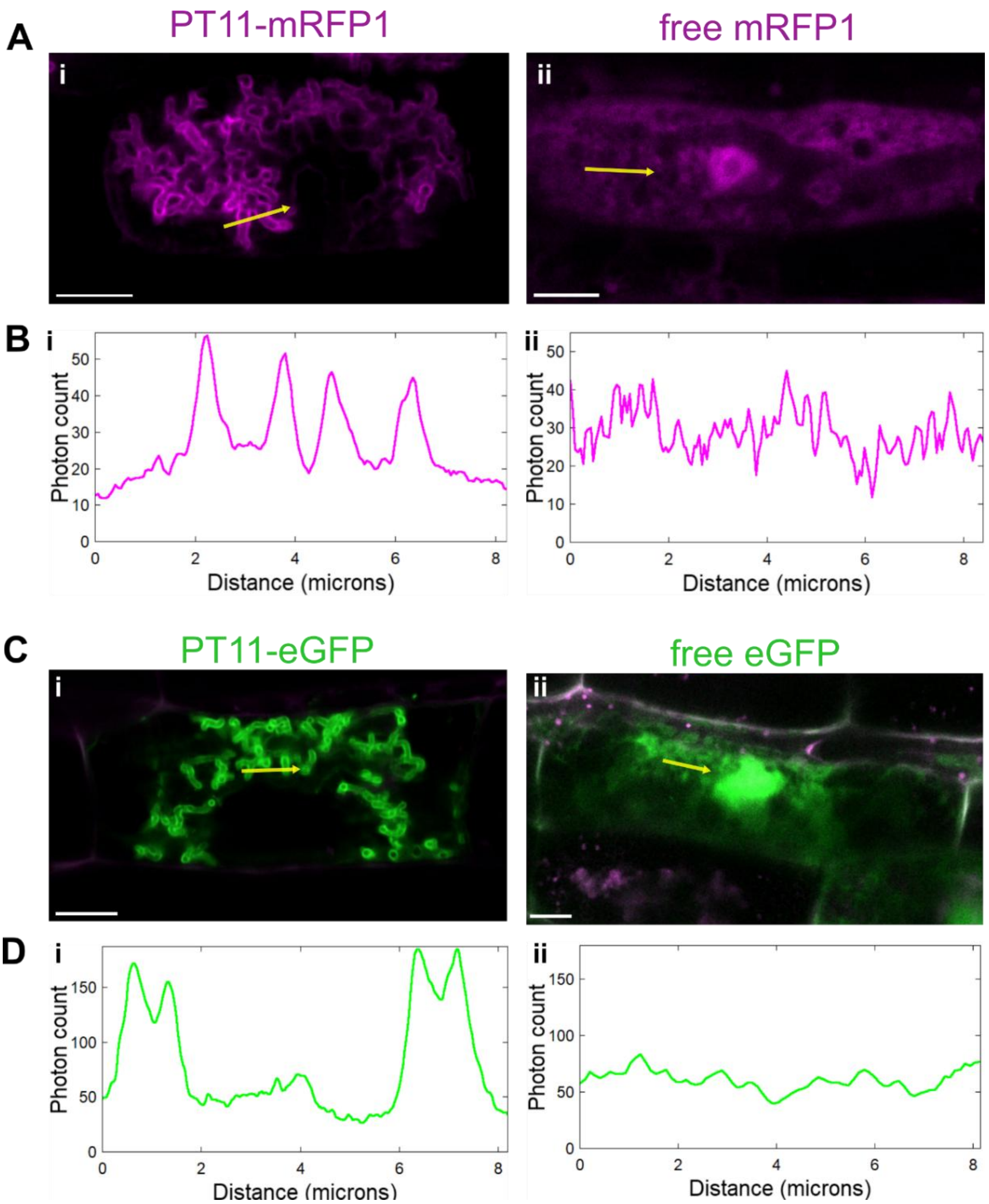


**Figure S7.** Localisation of GFP- and mRFP1-tagged PT11 compared to 'free' fluorescent proteins in arbusculated cells. Reporter rice plants were live imaged at 6 weeks post inoculation with *R. irregularis.* **A**) Representative images are shown of arbusculated cells in rice expressing mRFP1 either (i) tagged to PT11 (*pPT11:PT11-mRFP1*) or (ii) untagged (*pSTR2:mRFP1,* leading to 'free' mRFP1*),* with fluorescence intensity profiles across transects (yellow arrows) shown below in (Bi-ii). **C**) Representative images are shown of arbusculated cells in rice expressing eGFP either (i) tagged to PT11 (*pPT11:PT11-eGFP*) or (ii) untagged (*pSTR1:nls-eGFP,* ineffective NLS leads to 'free' eGFP*),* with fluorescence intensity profiles across transects (yellow arrows) shown below in **D**i-ii. Images are maximum intensity projections. Images are representative of observations made in 3+ independent experiments. Scale bars = 10 µm.

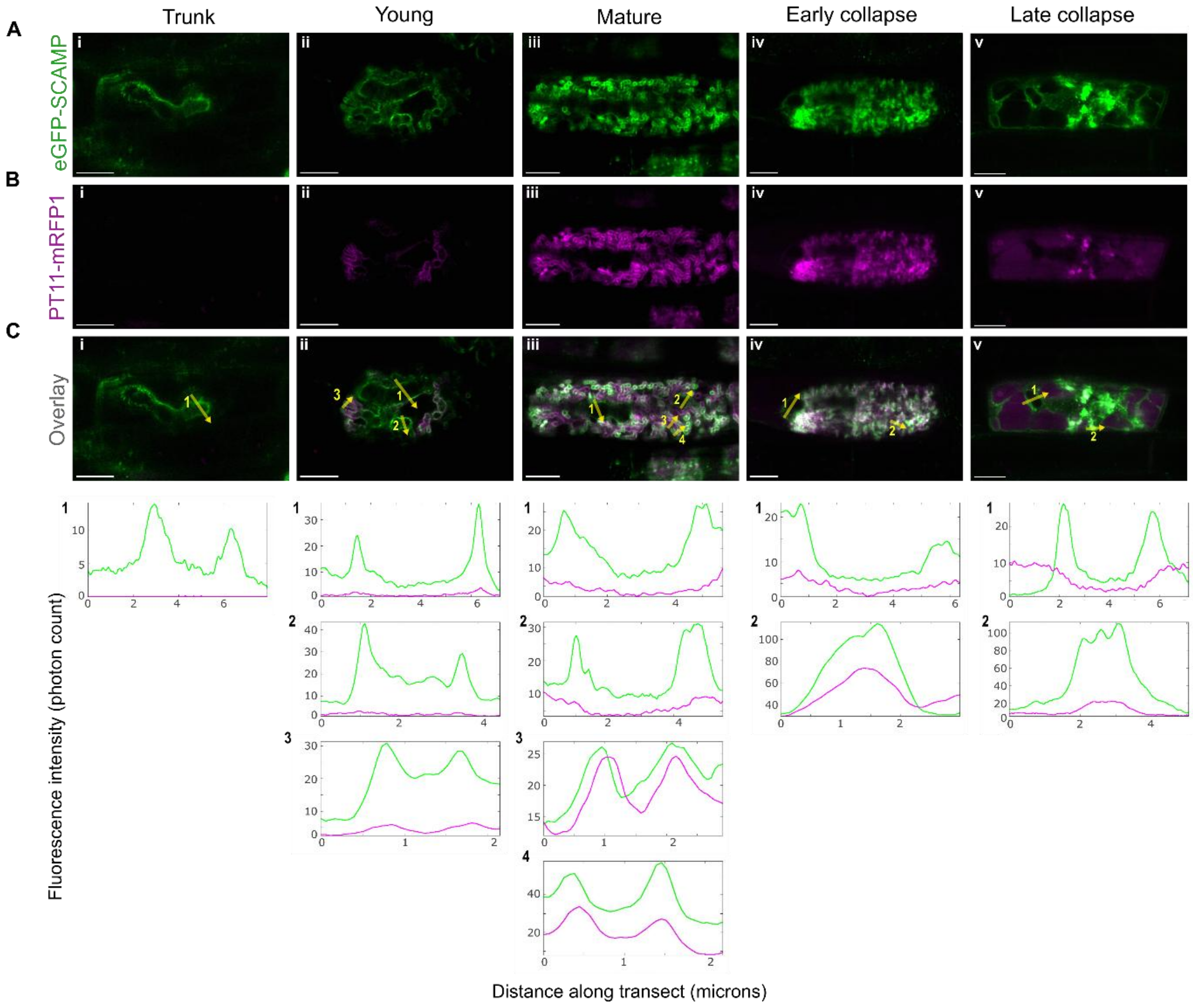


**Figure S8.** Relative abundance of PT11-mRFP1 and eGFP-SCAMP at different arbuscule domains. Rice plants co-expressing *pPT11:PT11-mRFP1* and *pSCAMP:eGFP-SCAMP* were live imaged at 6 weeks post inoculation with *R. irregularis.* Representative micrographs are shown of (i) trunk, (ii) young, (iii) mature, (iv) early collapse, and (v) late collapse arbuscules, with (**A**) eGFP-SCAMP channel, (**B**) PT11-mRFP1 channel, and (**C**) overlay (green = eGFP, magenta = mRFP1). Images are maximum intensity projections, scale bars = 10 µm. Fluorescence intensity plots for transects (yellow arrows) in **C**i-v are displayed below the corresponding micrographs. Green lines = eGFP intensity, magenta lines = mRFP1 intensity.

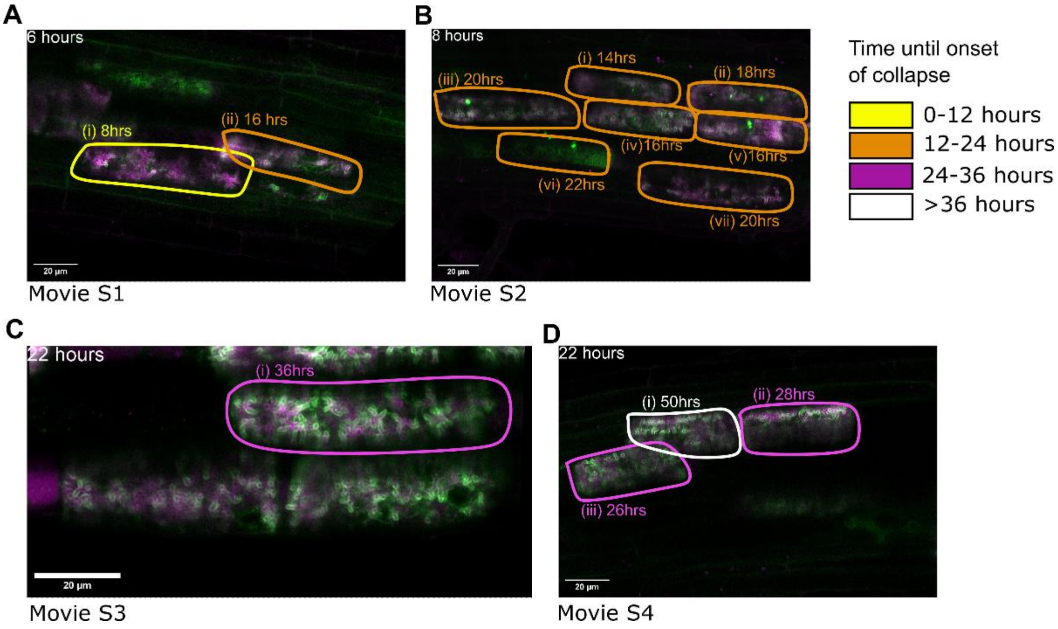


**Figure S9.** Accompaniment to Supplemental Movies 3-6 showing PT11-mRFP1 and eGFP-SCAMP dynamics at arbuscules with different lifespans. Arbuscules that were captured from their early development (trunk or first coarse branching) until their collapse are annotated, along with the hours taken until onset of collapse (yellow = 0-12 hours, orange = 12-24 hours, magenta = 24-36 hours, white = over 36 hours). Images are maximum intensity projections. Example timepoint selected for each timelapse movie to display all relevant arbuscules. Green = eGFP, magenta = mRFP1, scale bars = 20 µm.

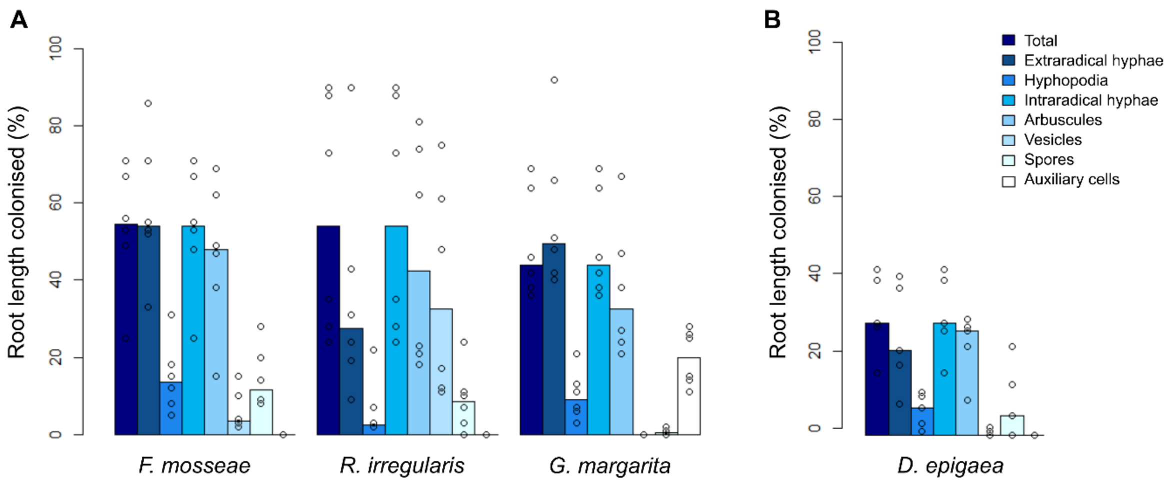


**Figure S10**. Arbuscular mycorrhizal colonisation quantification in rice colonised by diverse arbuscular mycorrhizal fungi. **A**) Root length colonisation quantification for rice at 6 weeks post inoculation with *Gigaspora margarita, Funneliformis mosseae,* or *Rhizophagus irregularis.* **B**) Root length colonisation quantification for rice at 6 weeks post inoculation with *Diversispora epigaea* (independent experiment from **A**). Graphs show percent of root hosting extraradical hyphae, hyphopodia, intraradical hyphae, arbuscules, vesicles, spores, auxiliary cells and any intraradical structure (Total), *n*= 6 plants per fungal species (**A**) or 5 plants (**B**). Raw data are available in the Source Data file.

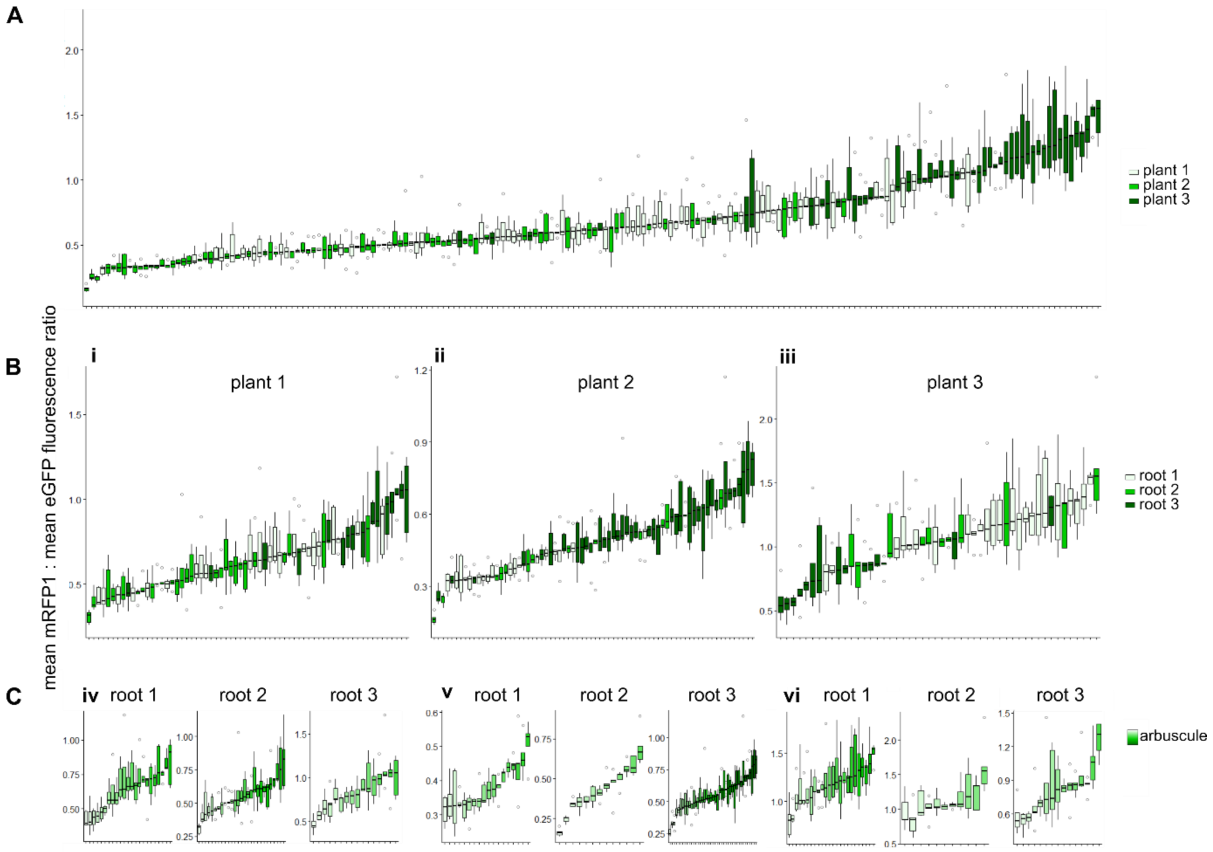


**Figure S11**. Variation in ratio of PT11 to SCAMP protein abundance at mature arbuscules of rice colonised by *R. irregularis.* Live imaging was performed on rice plants co-expressing *pPT11:PT11-mRFP1* and *pSCAMP:eGFP-SCAMP* at 6 weeks post inoculation. **A-C**) mRFP1:eGFP mean fluorescence intensity ratio was measured from 5 branch tips per arbuscule, all arbuscules of 1 colonisation zone per root, three roots per plant and three plant replicates. *n*= 66, 72 and 50 arbuscules for plant 1, 2 and 3, respectively. Data is ordered by median fluorescence ratio per arbuscule and displayed (**A**) ungrouped, where colour depicts plant replicates, (**B**) grouped by plant, where colour depicts root replicates, and (**C**) grouped by root. Plots show median and interquartile range (coloured box), data range excluding outliers (whiskers) and outliers (open circles, defined as datapoints outside 1.5* interquartile range). Raw data are available in the Source Data file.

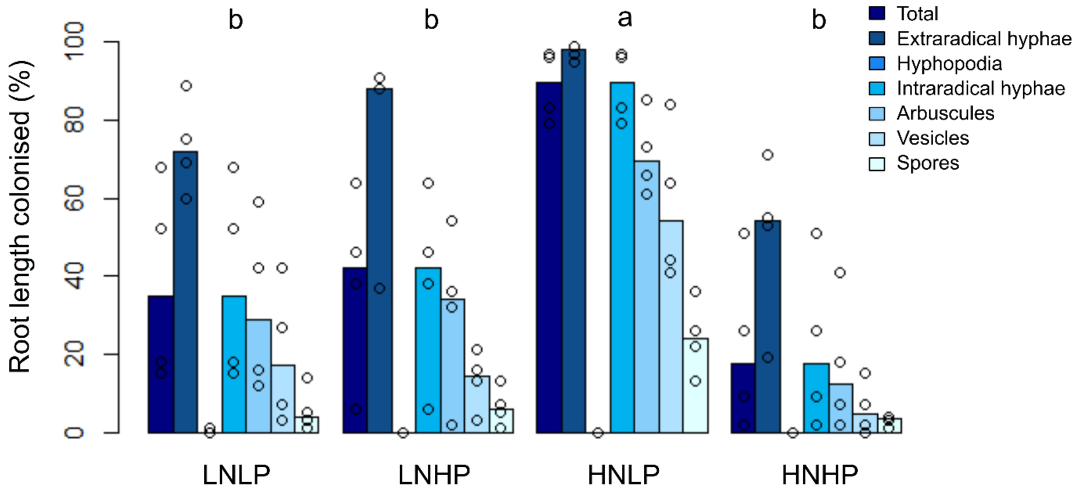


**Figure S12.** Arbuscular mycorrhizal colonisation of rice under different nutrient regimes. Colonisation of rice was quantified at 6 weeks post inoculation with *R. irregularis* after 4 weeks of high phosphate (HP, 250 µM) or low phosphate (LP, 25 µM) and high nitrate (HN, 3 mM) or low nitrate (LP, 0.05 mM) fertilisation. Graph shows percent of root hosting extraradical hyphae, hyphopodia, intraradical hyphae, arbuscules, vesicles, spores and any intraradical structure (Total). Letters depict result of Kruskall Wallis test on 'Total' colonisation level with Post-Hoc Dunn test ($p < 0.05$, $n = 4$ plants per nutrient treatment). Raw data are available in the Source Data file.

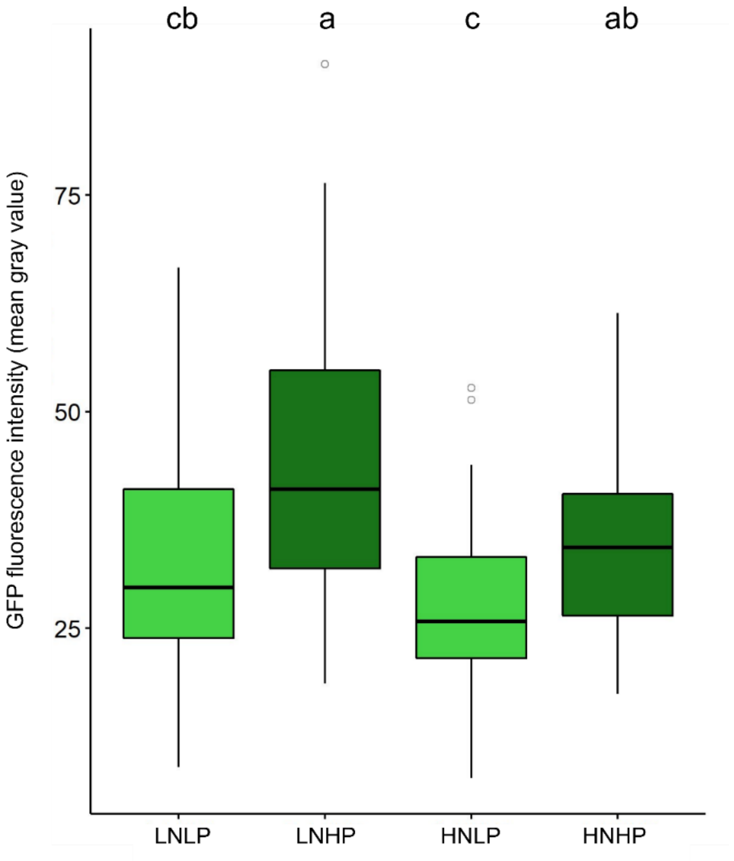


**Figure S13.** Abundance of eGFP-SCAMP at mature arbuscules of rice plants grown under differing nutrient regimes. The *pSCAMP:eGFP-SCAMP ; pPT11:PT11-mRFP1* co-expression rice line was live imaged at 6 weeks post inoculation with *R. irregularis* after 4 weeks of high phosphate (HP, 250 µM) or low phosphate (LP, 25 µM) and high nitrate (HN, 3 mM) or low nitrate (LP, 0.05 mM) fertilisation. Graph shows quantification of mean eGFP fluorescence intensity at mature arbuscules. Letters depict result of Kruskall Wallis test ($p = 1.36\times10^{-5}$) with Post-Hoc Dunn testing ($p < 0.05$). All arbuscules in one colonisation zone per root, three roots per plant, three plants per nutrient regime were imaged ($n$ = 34 (LNLP), 37 (LNHP), 39 (HNLP) and 33 (HNHP) arbuscule ). Plots show median and interquartile range (coloured box), data range excluding outliers (whiskers) and outliers (open circles, defined as datapoints outside 1.5* interquartile range). Raw data are available in the Source Data file.

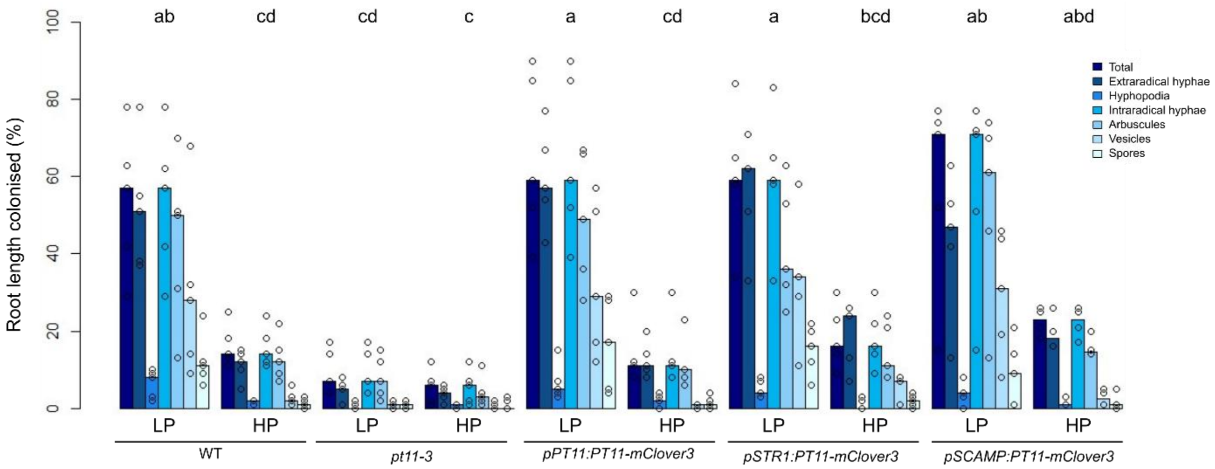


**Figure S14.** AM colonisation of PT11 promoter swap rice lines under high- and low-phosphate fertilisation. Colonisation of WT, *pt11-3,* and WT expressing *pPT11:PT11-mClover3, pSTR1:PT11-mClover3* or *pSCAMP:PT11-mClover3* was quantified at 6 weeks post inoculation with *R. irregularis* after 4 weeks of high phosphate (HP, 250 µM) or low phosphate (LP, 25 µM) fertilisation. Graph shows percent of root hosting extraradical hyphae, hyphopodia, intraradical hyphae, arbuscules, vesicles, spores and any intraradical structure (Total). Letters depict result of Kruskall Wallis test on 'Total' colonisation level with Post-Hoc Dunn test ($p < 0.05$, $n = 5$ plants per nutrient treatment). Raw data are available in the Source Data file.

**Table S1.** CRISPR small guide RNA sequences targeting *PT11* and genotyping primers

| | ***sgRNA1*** | ***sgRNA2*** |
|---|---|---|
| *PT11* CRISPR guides | GCGAGCAGTTTGGTGATCAG | TCATGGTTCCCCTTGTACGA |
| | | |
| | **Forward** | **Reverse** |
| *pt11-3* genotyping-by-sequencing | CATCCGCCAGTAGAAGGTCG | ACCAACGAAGGCCCACAACA |
| *pt11-4* genotyping-by-sequencing | TCACTGAGCTGAATTACACGC | CATCCGCCAGTAGAAGGTCG |

**Table S2.** Construct details for fluorescent reporter lines

| Construct | Promoter | N terminal | C terminal | Terminator | Selectable marker | Source |
|---|---|---|---|---|---|---|
| pGWB203-[GFP-AM42] | *pSCAMP (3kb)* | *eGFP* | *SCAMP* (genomic) | *tNos* | *HPT, NPTII* | (Kobae & Fujiwara, 2014) |
| pGWB204-[PT11-GFP] | *pPT11 (2.6kb)* | *PT11* (genomic) | *eGFP* | *tNos* | *HPT, NPTII* | (Kobae & Hata, 2010) |
| pJLD042 | *pPT11 (3kb)* | *NLS* | *TurboRFP* | *tAct2* | *HPT* | This work |
| pMSH19 | *pPT11 (3kb)* | *PT11 (CDS)* | *mRFP1* | *tAct2* | *HPT* | This work |
| pEW455 | *pSTR2* | *mRFP1* | | *tAct2* | *HPT* | This work |
| pMSH22 | *pSTR1* | *(nls-)eGFP* | | *tAct2* | *HPT* | This work |
| pEW452 | *pPT11 (3kb)* | *PT11 (CDS)* | *mClover3* | *tAct2* | *HPT* | This work. *mClover3* from Luginbuehl et al., (2020) |
| pEW453 | *pSTR1 (3kb)* | *PT11 (CDS)* | *mClover3* | *tAct2* | *HPT* | This work. *mClover3* from Luginbuehl et al., (2020) |
| pEW454 | *pSCAMP (3kb)* | *PT11 (CDS)* | *mClover3* | *tAct2* | *HPT* | This work. *mClover3* from Luginbuehl et al., (2020) |

**Table S3.** Genotyping primers used in this work

| Target amplicon | Forward primer | Reverse primer |
|---|---|---|
| *SCAMP* wild-type | GCCATATATGGCGATTCTTCGAG | AAGCGACATACTGCACTCGCTG |
| *scamp* mutant | CATCGGATGTCCAGTCCATTG | AAGCGACATACTGCACTCGCTG |
| *PT11* wild-type | CACGTTGTCCTGCTGACCCT | ACCAACGAAGGCCCACAACA |
| *pt11-3* mutant | CATCCGCCAGTAGAAGGTCG | ACCAACGAAGGCCCACAACA |
| *HPT* | GTTTATCGGCACTTTGCATCGGCCG | GATTTGTGTACGCCCGACAGTCC |
| *eGFP* | GTAAACGGCCACAAGTTCAG | TACAGCTCGTCCATGCCGAG |
| *TurboRFP* | GAAGGCACACAGACGATGAA | CTGTGGTCGACGAAATGAAATC |
| *mRFP1* | GTCATCAAGGAGTTCATGCG | CTCATCTTGATCTCGCCCTT |
| *PT11-mClover3* | TTCATGCTGGCGATGGGCATCC | TGCTGCTTCATGTGGTCAGG |

**Table S4.** Laser and detector settings used in this work

| | Excitation wavelength (nm) | Fluorophore emission range (nm) | Autofluorescence emission range (nm) |
|---|---|---|---|
| eGFP | 488 (WLL) | 500-550 (HyD) | 600-650 (HyD) |
| mRFP1 | 584 (WLL) | 600-650 (HyD) | 700-750 (HyD) |
| mClover3 | 506 (WLL) | 515-565 (HyD) | 615-665 (HyD) |
| Alexafluor$^{488}$ | 488 (WLL) | 500-550 (HyD) | |
| Propidium iodide | 488 (WLL) | 600-650 (HyD) | |

**Table S5.** Recipe for Rice Half Hoagland's fertiliser solution

| **Macronutrient** | **Concentration (mM)** |
|---|---|
| $KNO_3$ | 2.500 |
| $Ca(NO_3)_2.4H_2O$ | 0.250 |
| $MgSO_4.7H_2O$ | 1.000 |
| $KH_2PO_4$ | 0.025 |
| KCL | 0.475 |
| Fe-Citrate | 0.010 |
| | |
| **Micronutrients** | **Concentration (µM)** |
| $MnSO_4.H_2O$ | 4.082 |
| $ZnSO_4.7H_2O$ | 0.348 |
| $CuSO_4.5H_2O$ | 0.160 |
| $Na_2B_4O_7.10H_2O$ | 2.622 |
| $(NH_4)_6Mo_7O_{24}.4H_2O$ | 0.040 |

**Table S6.** Amendments to Half Hoagland's fertiliser solution for nutrient regime experiments

| **Component** | **Concentration (mM)** | | | |
|---|---|---|---|---|
| | **HNLP** | **HNHP** | **LNHP** | **LNLP** |
| $KNO_3$ | 2.500 | 2.500 | 0.000 | 0.000 |
| $Ca(NO_3)_2.4H_2O$ | 0.250 | 0.250 | 0.025 | 0.025 |
| $KH_2PO_4$ | 0.025 | 0.250 | 0.25 | 0.025 |
| KCL | 0.475 | 0.250 | 2.75 | 2.975 |
| $CaCl_2$ | 1.000 | 1.000 | 1.225 | 1.225 |
