# Supplementary material for: Symbiotic phosphate transporter dynamics in rice expose functional plasticity of the arbuscules
Source: Nat Commun. 2026 Apr 22;17:5450. doi: 10.1038/s41467-026-71496-8 (PMC13282384; doi:10.1038/s41467-026-71496-8)
Supplement: Supplementary file 2 — Description of Additional Supplementary Files [file 41467_2026_71496_MOESM2_ESM.pdf]

Description of Additional Supplementary Files

File Name: Supplementary Movie 1

Description: Timelapse movie of arbuscule development and collapse in rice, showing diverse arbuscule developmental trajectories and lifespans. *pSCAMP:eGFP-SCAMP* reporter rice plantwas colonised by *R. irregularis* in an AMSlide and imaged non-invasively at 2-hour intervals. Images are maximum intensity z projections. Green = eGFP, scale bar = 10 µm. Arbuscules and their developmental stages are highlighted in Supplemental Fig. S2.

File Name: Supplementary Movie 2

Description: Timelapse movie showing PT11 dynamics throughout arbuscule development and collapse. *pSCAMP:eGFP-SCAMP ; pPT11:PT11-mRFP1* co-expression rice line was colonised by *R. irregularis* in an AMSlide and imaged non-invasively at 2-hour intervals. Images are maximum intensity z projections. Green = eGFP, magenta = mRFP1, scale bar = 10 µm.

File Name: Supplementary Movie 3

Description: Timelapse of PT11-mRFP1 and eGFP-SCAMP dynamics in arbuscules that collapse after 8-16 hours. *pSCAMP:eGFP-SCAMP ; pPT11:PT11-mRFP1* co-expression rice line colonised by *R. irregularis* live imaged in AMSlide3. Images taken at 2-hour intervals over 22 hours. Green = eGFP, magenta = mRFP1 (and autofluorescence). Scale bar = 20 µm. Arbuscules and lifetimes annotated in Supplemental Fig. S9A.

File Name: Supplementary Movie 4

Description: Timelapse of PT11-mRFP1 and eGFP-SCAMP dynamics in arbuscules that collapse after 14-22 hours. *pSCAMP:eGFP-SCAMP ; pPT11:PT11-mRFP1* co-expression rice line colonised by *R. irregularis* live imaged in AMSlide3. Images taken at 2-hour intervals over 28 hours. Green = eGFP, magenta = mRFP1 (and autofluorescence). Scale bar = 20 µm. Arbuscules and lifetimes annotated in Supplemental Fig. S9B.

File Name: Supplementary Movie 5

Description: Timelapse of PT11-mRFP1 and eGFP-SCAMP dynamics in an arbuscule that collapses after 36 hours. *pSCAMP:eGFP-SCAMP ; pPT11:PT11-mRFP1* co-expression rice line colonised by *R. irregularis* live imaged in AMSlide3. Images taken

at 2-hour intervals over 52 hours. Green = eGFP, magenta = mRFP1 (and autofluorescence). Scale bar = 20 µm. Arbuscules and lifetimes annotated in Supplemental Fig. S9C.

File Name: Supplementary Movie 6

Description: Timelapse of PT11-mRFP1 and eGFP-SCAMP dynamics in arbuscules that collapse after 26-50 hours. *pSCAMP:eGFP-SCAMP ; pPT11:PT11-mRFP1* co-expression rice line colonised by *R. irregularis* live imaged in AMSlide3. Images taken at 2-hour intervals over 52 hours. Green = eGFP, magenta = mRFP1 (and autofluorescence). Scale bar = 20 µm. Arbuscules and lifetimes annotated in Supplemental Fig. S9D.
