# Supplementary material for: Symbiotic phosphate transporter dynamics in rice expose functional plasticity of the arbuscules
Source: Nat Commun. 2026 Apr 22;17:5450. doi: 10.1038/s41467-026-71496-8 (PMC13282384; doi:10.1038/s41467-026-71496-8)
Supplement: Supplementary file 9 — Reporting Summary [file 41467_2026_71496_MOESM9_ESM.pdf]

Corresponding author(s): Uta Paszkowski and Jennifer McGaley

Last updated by author(s): Mar 10, 2026

Reporting Summary

Nature Portfolio wishes to improve the reproducibility of the work that we publish. This form provides structure for consistency and transparency in reporting. For further information on Nature Portfolio policies, see our Editorial Policies and the Editorial Policy Checklist.

Statistics

For all statistical analyses, confirm that the following items are present in the figure legend, table legend, main text, or Methods section.

n/a	Confirmed	
☐	☒	The exact sample size (n) for each experimental group/condition, given as a discrete number and unit of measurement
☐	☒	A statement on whether measurements were taken from distinct samples or whether the same sample was measured repeatedly
☐	☒	The statistical test(s) used AND whether they are one- or two-sided<br>*Only common tests should be described solely by name; describe more complex techniques in the Methods section.*
☒	☐	A description of all covariates tested
☐	☒	A description of any assumptions or corrections, such as tests of normality and adjustment for multiple comparisons
☐	☒	A full description of the statistical parameters including central tendency (e.g. means) or other basic estimates (e.g. regression coefficient) AND variation (e.g. standard deviation) or associated estimates of uncertainty (e.g. confidence intervals)
☐	☒	For null hypothesis testing, the test statistic (e.g. F, t, r) with confidence intervals, effect sizes, degrees of freedom and P value noted<br>*Give P values as exact values whenever suitable.*
☒	☐	For Bayesian analysis, information on the choice of priors and Markov chain Monte Carlo settings
☒	☐	For hierarchical and complex designs, identification of the appropriate level for tests and full reporting of outcomes
☒	☐	Estimates of effect sizes (e.g. Cohen's d, Pearson's r), indicating how they were calculated

Our web collection on statistics for biologists contains articles on many of the points above.

Software and code

Policy information about availability of computer code

Data collection	FIJI/ImageJ was used to collect data in this study. Version: FIJI/ImageJ 2.16.0/1.54p. Schindelin et al., (2012). Fiji: An open-source platform for biological-image analysis. Nature Methods, 9(7), Article 7
Data analysis	FIJI/ImageJ was used to analyze data in this study. Version: FIJI/ImageJ 2.16.0/1.54p. Schindelin et al., (2012). Fiji: An open-source platform for biological-image analysis. Nature Methods, 9(7), Article 7.<br><br>Base R was used to analyze data in this study. Version: R 4.5.0. R Core Team. (2024). R: A Language and Environment for Statistical Computing [Computer software]. R Foundation for Statistical Computing. https://www.R-project.org/<br><br>The R package ggplot2 was used to analyze data in this study. Wickham, H. (2016). ggplot2: Elegant Graphics for Data Analysis. Springer International Publishing.<br><br>The R package lme4 was used to analyze data in this study. Bates et al., (2015). Fitting Linear Mixed-Effects Models Using lme4. Journal of Statistical Software, 67, 1–48.

For manuscripts utilizing custom algorithms or software that are central to the research but not yet described in published literature, software must be made available to editors and reviewers. We strongly encourage code deposition in a community repository (e.g. GitHub). See the Nature Portfolio guidelines for submitting code & software for further information.

Data

Policy information about availability of data

All manuscripts must include a data availability statement. This statement should provide the following information, where applicable:

- Accession codes, unique identifiers, or web links for publicly available datasets
- A description of any restrictions on data availability
- For clinical datasets or third party data, please ensure that the statement adheres to our policy

All data supporting the findings of this study are available within the paper and its Supplementary Information. Identifiers of all genes involved in this study are listed under 'Accession numbers' and refer to the Rice Genome Annotation Project Database (https://rice.uga.edu/). All primer sequences are listed in Tables S1 and S3. All construct identifiers and their sources are listed in Table S2. Constructs and plant materials produced in this work are available upon request to the corresponding author.

Research involving human participants, their data, or biological material

Policy information about studies with human participants or human data. See also policy information about sex, gender (identity/presentation), and sexual orientation and race, ethnicity and racism.

Reporting on sex and gender	Not applicable to this study.
Reporting on race, ethnicity, or other socially relevant groupings	Not applicable to this study.
Population characteristics	Not applicable to this study.
Recruitment	Not applicable to this study.
Ethics oversight	Not applicable to this study.

Note that full information on the approval of the study protocol must also be provided in the manuscript.

Field-specific reporting

Please select the one below that is the best fit for your research. If you are not sure, read the appropriate sections before making your selection.

[x] Life sciences [ ] Behavioural & social sciences [ ] Ecological, evolutionary & environmental sciences

For a reference copy of the document with all sections, see nature.com/documents/nr-reporting-summary-flat.pdf

Life sciences study design

All studies must disclose on these points even when the disclosure is negative.

Sample size

Live imaging experiments (fluorescence intensity measurements): a restricted maximum likelihood analysis of mixed effect models was carried out in this work to identify at which hierarchical level variance exists and to structure sampling accordingly. In this system, most variance occurs at the inter-root and inter-arbuscule level, not at the inter-plant level ('Results', Fig. 5). Due to this finding, and the fact that arbuscules co-exist in a continuum of development, data was collected from all relevant (e.g. mature stage) arbuscules of one colonisation zone per root, three roots per plant and a minimum of three plants per genotype or treatment (detailed in 'Methods' section and respective figure legends). Further replicates were not feasible due to the extensive time required for imaging and necessity to perform all imaging within a restricted timeframe because the samples are live.

Timelapse experiments: due to the time-consuming nature of performing manual timelapse experiments, the replicate number was constrained by the imaging time available at/between each timepoint. For 24-hour interval timelapses, 56 arbuscules from 5 plants were imaged. This was sufficient to reveal 4 main developmental trajectories, with multiple arbuscules showing each trajectory. For 2-hour interval timelapses, the selected timelapses are representative of observations made from three different plants over multiple times of day, days, and weeks. Manual imaging overnight constrained further replicates, but results corroborate findings from the 24-hour interval timelapses and previous reports (McGaley et al., 2024, Kobae & Fujiwara, 2014, Kobae et al., 2016).

Colonisation assessment: as described in the methods (Roth et al., 2018), ten points were analysed per root, ten roots per plant, four-to-six plants per experiment depending on the scale of the experiment and feasibility of higher replicate numbers. Most colonisation experiments in this study aimed to detect the effect of Ospt11 mutation on mycorrhizal colonisation, and its rescue (or lack of) by fluorescent reporter constructs. Previously, three biological replicates was reported to be sufficient to detect reduced colonisation in rice Ospt11 mutants (Yang et al., 2012). More replicates were included here to resolve finer phenotypic differences. Where arbuscule morphology was assessed, the presented images are representative of observations of three plant replicates.

Arbuscule size measurements: previously, 24 arbuscules per plant, three plants per genotype were reported to be sufficient to detect the arbuscule phenotype of Ospt11 mutants in rice (Yang et al., 2012). To better resolve effects of a new pt11 mutant allele in this study, and considering the high inter-arbuscule variance measured (see above), 50 arbuscules per plant were measured, with three plants per genotype.

Data exclusions	No data were excluded from the analyses.
Replication	Live imaging experiments and colonisation assessment: all data presented here corroborates observations made in at least one independent experiment. All data replicates smaller-scale observations made in former generations of transgenic reporter lines and mutant lines. In instances of transgene silencing (loss of fluorescent reporter expression in a line/generation), a different independent reporter line was used and transgene expression (fluorescence) confirmed.<br><br>Timelapse experiments: timelapse experiments were performed at different temporal scales over multiple years of experimentation. The data presented here are representative of these observations.<br><br>All attempts at replication were therefore successful, verifying reproducibility of the results.
Randomization	Plant replicates were randomly distributed between experimental groups (e.g. nutrient treatments) and randomly arranged within growth set-ups to avoid effects of covariates.
Blinding	Live imaging experiments (fluorescence intensity measurements): no blinding was necessary. During imaging, detector channel LUT was auto-scaled such that any differences in fluorescence intensity were not detectable by eye. The same approach was taken during fluorescence intensity measurements.<br><br>Timelapse experiments: no blinding necessary (no groups tested).<br><br>Colonisation assessment: investigators assessed colonisation blind.<br><br>Arbuscule size measurements: blinding was not performed, as images were taken of all arbuscules in a plant (until 50 replicates achieved) and perimeters of all arbuscules were measured without exception.

Reporting for specific materials, systems and methods

We require information from authors about some types of materials, experimental systems and methods used in many studies. Here, indicate whether each material, system or method listed is relevant to your study. If you are not sure if a list item applies to your research, read the appropriate section before selecting a response.

Materials & experimental systems

n/a	Involved in the study
☒	☐ Antibodies
☒	☐ Eukaryotic cell lines
☒	☐ Palaeontology and archaeology
☒	☐ Animals and other organisms
☒	☐ Clinical data
☒	☐ Dual use research of concern
☐	☒ Plants

Methods

n/a	Involved in the study
☒	☐ ChIP-seq
☒	☐ Flow cytometry
☒	☐ MRI-based neuroimaging

Dual use research of concern

Policy information about dual use research of concern

Hazards

Could the accidental, deliberate or reckless misuse of agents or technologies generated in the work, or the application of information presented in the manuscript, pose a threat to:

No	Yes
☒	☐ Public health
☒	☐ National security
☒	☐ Crops and/or livestock
☒	☐ Ecosystems
☒	☐ Any other significant area

Experiments of concern

Does the work involve any of these experiments of concern:

No	Yes	
☒	☐	Demonstrate how to render a vaccine ineffective
☒	☐	Confer resistance to therapeutically useful antibiotics or antiviral agents
☒	☐	Enhance the virulence of a pathogen or render a nonpathogen virulent
☒	☐	Increase transmissibility of a pathogen
☒	☐	Alter the host range of a pathogen
☒	☐	Enable evasion of diagnostic/detection modalities
☒	☐	Enable the weaponization of a biological agent or toxin
☒	☐	Any other potentially harmful combination of experiments and agents

Plants

Seed stocks

Sources of all rice lines are reported in 'Methods' section:
- pSCAMP:eGFP-SCAMP and pPT11:PT11-eGFP from Yoshihiro Kobae lab, Japan. Production reported in Kobae & Fujiwara (2014) and Kobae & Hata (2010).
- All other genetic material produced in this work, with seed produced in contained glasshouse environment in Cambridge, UK and available upon request to corresponding author.

Sources of all fungal isolates are reported in 'Methods', with isolate codes listed, according to the Swiss Collection of Arbuscular Mycorrhizal Fungi and International Bank for the Glomeromycota.

Novel plant genotypes

Transgenic reporter lines: the methods by which new transgenic reporter lines were produced are reported in the 'Methods' section. In summary, transgenes were introduced into the rice genome by Agrobacterium-mediated transformation. Where enough transformed rice plants were acquired, at least three regenerated transformants were assessed at the T0 stage to ensure similar phenotype (i.e. ensuring no effects of tissue culture/transgene insertion position). The experiments reported in this study were all performed on T2+ generation plants.

Mutant lines: all new mutant lines were generated by CRISPR Cas9 gene editing, using the Csy-type ribonuclease 4 ribozyme approach as detailed in the 'Methods' section. In summary, two small guide RNA sequences were designed to target the endogenous gene sequence, spanning the promoter and first exon (OsPT11, LOC_Os01g46860). The Cermak et al., (2017) toolkit was used to assemble small guide RNAs into the Cas9-containing vector for Agrobacterium-mediated transformation into rice. Two independent transformant lines (homozygous, Cas9-free) were assessed to ensure any phenotypes were not the result of tissue culture or off target mutations. By crossing to fluorescent reporter lines, any background effects were also removed. All experiments reported in this study were performed on T2+ generation plants.

Authentication

All newly generated transgenic reporter lines were authenticated by PCR genotyping using the primers listed in Table S3. Multiple independent primary transformant lines were screened to ensure similar fluorescence distributions and plant/fungal colonisation phenotypes were observed (i.e. phenotypes not caused by secondary effects of transgene insertion). To avoid effects of mosaicism, experiments in this study were only performed in T2+ generation stable transformants. Many rporter lines underwent genetic crossing and retained the same phenotype regardless of background landscape.

New mutants generated via CRISPR Cas9 editing (pt11-3, pt11-4) were authenticated by Sanger Sequencing of target edit site. The Cas9-containing transgene was segregated out of all experimental lines. Two independent CRISPR alleles were assessed to ensure phenotypes were not result of off-target effects. To avoid effects of mosaicism, experiments in this study were only performed in T2+ generation. Similar phenotypes were observed in mutant lines that had been crossed to fluorescent reporter line negative controls, simulating a backcross (e.g. pPT11:NLS-TurboRFP in pt11-3).
