# Supplementary material for: Symbiotic phosphate transporter dynamics in rice expose functional plasticity of the arbuscules
Source: Nat Commun. 2026 Apr 22;17:5450. doi: 10.1038/s41467-026-71496-8 (PMC13282384; doi:10.1038/s41467-026-71496-8)
Supplement: Supplementary file 10 — Transparent Peer Review file [file 41467_2026_71496_MOESM10_ESM.pdf]

nature portfolio

Peer Review File

Symbiotic phosphate transporter dynamics in rice expose functional plasticity of the arbuscules

Corresponding Author: Dr Uta Paszkowski

Parts of this Peer Review File have been redacted as indicated to remove third-party material.

This file contains all reviewer reports in order by version, followed by all author rebuttals in order by version.

Version 0:

Reviewer comments:

Reviewer #1

(Remarks to the Author)
The study by Mac Galey and colleagues presents an unprecedented insight into the cellular-level dynamics of arbuscular mycorrhizal symbiosis. By combining innovative non-invasive live imaging with elegant genetic approaches, the authors provide the most detailed temporal and spatial characterization to date of arbuscule development, collapse, and phosphate transporter dynamics. The discovery that arbuscules are not identical units of nutrient exchange, but display heterogeneity in lifespan, developmental trajectories, and PT11 abundance, is highly novel and of broad significance. These findings have profound implications for how we interpret AM colonization and evaluate symbiotic efficiency. Lastly, the use of different AM fungi with diverse colonization patterns and solid controls such as mutant phenotype complementation with the fluorescent protein fusion constructs give broad and sound support to the Author conclusions.

I have noted a few minor points that may be improved:
- While the reference list is extensive and covers most of the seminal literature, it would be worth double-checking whether all recent advances in single-cell or spatial transcriptomics in AM symbiosis have been cited, as these relate directly to the emerging concept of heterogeneity among arbusculated cells.

- The manuscript is generally well written, but in some sections (especially Results), the density of detail occasionally hampers readability. The Authors might consider shortening or restructuring very long sentences to aid comprehension.
- the legend of figure 1A should include the indication of mid-grey color for the interface compartment

- Terms such as “collapse,” “degeneration,” and “senescence” are sometimes used interchangeably for arbuscule decline. A consistent terminology might improve clarity, unless different meanings are intentionally used.

Reviewer #2

(Remarks to the Author)
In the manuscript titled “Symbiotic phosphate transporter dynamics in rice expose functional plasticity of the arbuscules,” McGaley et al. utilize stable transgenic lines of rice expressing fluorescent protein–tagged phosphate transporter PT11 to track and describe its presence on the periarbuscular membrane (PAM) throughout arbuscule development during mutualistic symbiotic interaction with arbuscular mycorrhizal (AM) fungi. The presented work is primarily descriptive (which is not a negative characterization) and provides remarkable new details through non-invasive imaging of fluorescently tagged reporters in colonized rice roots. Considering the inherent difficulties of such imaging, the effort and work are tremendous, and the primary author should be recognized and praised. The measurements of arbuscule lifespan are important and deserve great attention.
However, the manuscript lacks adequate interpretation (and presentation) of the obtained data and suffers from exaggerated and embellished language, which diminishes the impact of the results. Moreover, more than half of the data (Figures 3 to 6) are based on the inferior fluorescent PT11-mRFP reporter, whereas the superior reporters PT11-GFP and PT11-mClover3 were used only as supplementary.
The title and the summary paragraph of the introduction (lines 67–80) exemplify exaggerated language and interpretations

that are not supported by the data. There is no evidence supporting the claim of “functional plasticity of the arbuscules” (title) or the statement “…uncovered a cellular-level of nutrient regulation of symbiotic phosphate uptake critical to symbiotic success. The observed capacity for symbiotic nutrient exchange to be fine-tuned at the arbuscule level” (lines 74–76). The presented work only demonstrates the fluorescence of PT11-FP at different stages of development and different conditions and does not show actual phosphate transport at any stage of arbuscule development to justify such statements.
Figure 1
It is emphasized that SCAMP-GFP accumulates at branch tips at stages ii and iii (Fig. 1B). Such accumulation is not visible. Why is it important that SCAMP-GFP accumulates at fine branches, given that it is a marker of the plasma membrane and PAM?
Supplemental Fig. S2A–H is beautiful and informative and should be presented as a main figure along with the data in Fig. 1C.
It is unclear why the term “coarse” arbuscule branches is introduced instead of the commonly used “thick” branches. This change should be clarified and explained.
Figure 2
SCAMP-GFP is a marker of the plasma membrane and PAM. However, it is very difficult to see well-defined labeling of these two membranes in the presented images. One must assume it is on the membranes. While it is understandable that obtaining high-quality images is challenging, the images presented should be clear and representative. Such clear presentation is especially important for newcomers in the field who are learning about the cell biology of AM symbiosis.
Figure 3
This figure introduces a new fluorescent reporter of PT11 as a fusion to mRFP and describes the creation of a rice stable transgenic line co-expressing PT11-mRFP and GFP-SCAMP for co-localization studies. This transgenic line was used to assess PT11-mRFP and GFP-SCAMP location at different stages of arbuscule development. However, PT11-mRFP appears to be a highly inferior reporter, as it is impossible to see it on the PAM; the signal is visible primarily as cytoplasmic and vacuolar haze (Fig. 3B). It is puzzling that the manuscript states PT11-mRFP locates on the PAM similarly to the PT11-GFP reporter (Fig. 3A). There is no similarity in the fluorescent pattern, and it is misleading to claim this (lines 160–161).
Figures 3C and S6 present simultaneous localization of PT11-mRFP and GFP-SCAMP or PT11-mRFP and PT11-GFP, respectively. Separate images showing only PT11-mRFP fluorescence must be presented in addition to the overlay images in both figures.
Lines 176–177: “…but all PT11 was removed from the PAM at collapsed arbuscules.” How was the removal of PT11 monitored? This should be clarified.
Figure 4
The PT11-mRFP reporter was used to monitor its location at different stages of arbuscule development from different species of AM fungi. Similar to the previous figure, it is difficult to see PT11-mRFP on the PAM; the signal appears mainly as fluorescent haze. The statement “PT11 was still visible only in cells hosting young and mature arbuscules…” (lines 202–203) is insufficient and incorrect, as fluorescence from PT11-mRFP (magenta) is shown. PT11 should be on the PAM, which is not evident in the presented images.
Images in Fig. 4E are particularly inadequate, as it is almost impossible to discern anything in any light channel.
Figure 5
The measurements presented in Fig. 5 are based on the use of the “poor” PT11-mRFP reporter (see above). In the corresponding section of the manuscript (lines 210–223), it is concluded that PT11 shows high variability in abundance on the PAM. It is unclear how such a conclusion can be made, given that no clear evidence has been presented showing PT11-mRFP localization on the PAM.
Figure 6
Similar comments apply to the data in Fig. 6. Higher PT11-mRFP intensities are detected under lower phosphate conditions (line 239). Were these higher intensities detected on the PAM? This is unclear from the images.
In the corresponding section of the manuscript (lines 226–249), the word “confirming” is used extensively to describe the data. This could indicate confirmational bias rather than critical interpretation of the results.
Figure 7
This is the best data in the entire manuscript. The fluorescent images of PT11-mClover3 are beautiful and high-quality, with clear localization of the reporter on the PAM. It is puzzling why this reporter was not used in all analyses presented in the previous figures. Additionally, the figure legend is highly informative and detailed, in contrast to the legends for earlier figures. In general, Fig. 7 should be the first figure of the manuscript.
The data in Fig. 7B are robust and informative, in contrast to the data in Fig. 6B, which show only a minuscule difference in fluorescence intensities. Fig. 7 uses “mean photon count,” whereas Fig. 6B uses “mean gray value.” Why were different metrics used? This should be explained and added to the figure legend.
Line 245-249. It seems that this sentence is not complete.
Line 293-295. What this sentence mean?

Reviewer #3

(Remarks to the Author)
Manuscript of McGaley et al.

General comments
This manuscript focuses on the study of important root symbiosis between plants and arbuscular mycorrhizal (AM) fungi, forming specialized hyphal structures known as arbuscules, for nutrient transfer (particularly phosphate) to the host plant. In this work, the authors employed live cell imaging methods along with validated fluorescent markers (the membrane marker SCAMP and PT11 phosphate transporter, as a proxi of P transport) to investigate the developmental trajectory of arbuscules

and PT11 transcription/protein localization at cellular resolution. This research provides novel insights into (i) the dynamic phases of arbuscule growth and collapse within individual rice cells; (ii) the localization of PT11 at specific stages of arbuscule development across different AM fungal interactions; and (iii) the cell-specific regulation of PT11 abundance in individual arbuscules, which the authors demonstrate to be under nutrient (P) regulation.
The results are based on appropriate methodologies and tools, producing high-quality data that are generally well supported by robust analysis. Overall, the findings are compelling and contribute to our understanding of the cellular steps underlying arbuscule development and collapse and the contribution of P11 contribution to this process. Most of the analyses rely on the use of SCAMP and PT11, which have been previously validated as useful markers for studying arbuscule development in rice. The novelty here lies in their use in fine cellular resolution studies, including original colocalization experiments, producing new descriptive and quantitative data on developmental trajectories of individual arbuscules, in addition to providing cellular information on PT11 promoter activity and protein localization in branched arbuscule stage. The conclusions drawn in these sections are sound and well supported by the data, although improvements to figure descriptions and labelling—particularly to assist non-specialist readers—are required to enhance clarity (as outlined below).
The manuscript also presents robust data on the cell-autonomous regulation of PTR11 abundance in individual cells and clearly demonstrates that this abundance is influenced by nutrient (P) conditions. This provides evidence for a cellular layer of nutrient regulation, which is very interesting. However, I have a concern about the overstatement that this change in abundance directly reflects the regulation of nutrient transport, which may be the case, but this is not what the data actually show. Such a conclusion requires additional supporting evidence (outlined below).

Specific comments:
-Line 67. PT11 is used as a proxi of P transport, however, the rationale behind using PT11 rather than another P transporter should be explained. To what extent can general conclusions about P transport be drawn only on the basis of P11? Would it not have been relevant to at least compare the dynamics of PT11 with those of another P transporter?
-Line 87: Many experiments in the manuscript rely on the use of SCAMP and PT11, used in previous studies, yet background information on these markers is not well developed in the text. Please provide this background information to better highlight what is new here relative to these previous studies.
-Fig. 1: The data description is not always accessible to non-specialists. It is not always obvious what is being described in the fluorescence images. Bright field images should be included to visualise cell contours and arbuscules. Label the diverse structures in the images (e.g. coarse and fine branches, trunk, etc.). Despite the arrowheads, transvacuolar strands are not clearly visible either. 'Cb' refers to both coarse branches and collapsing branches (please modify this). Clarify the labelling in the other figures as well.
-Lines 108-112: 15% of arbuscules collapse quickly. How consistent is this number across different experiments? Could the collapse be triggered by the experimental conditions, which are based on repeated observations of samples using confocal microscopy? A control experiment without repeated observations could help determine the biological significance of this variation.
-Line 217: The inter-root-level variations in PT11 abundance should be better explained. Providing information on root features (size and diameter), developmental stage (growing region or older part of roots) or root type (main vs adventitious vs lateral roots?) from where the arbuscule data was extracted could help understanding it.
-Line 225: From the data shown in Fig 7, it is concluded that the abundance in PT11 protein fusion levels is variable in different LP vs HP nutrient conditions. This is indeed a coherent conclusion, reflecting what the data really shows. However, in this section and throughout the manuscript, there is an overstatement of this conclusion, with 'functional' inferences about P transport being drawn from this data. It is important to remove this ambigous statetement from the text. Additional experimental proof is also needed to understand the significance of these cell-specific variations in PT11 levels. First, it is important to verify if variations in PT11 levels seen in LP/HP conditions is not simply reflecting fine-transcriptional regulation of PT11 promoter that is driving fusion expression. Using the PT11 transcriptional fusion (Fig 2) or in co-dynamic studies with the P11 protein fusion in LN and HN conditions, can help determining that. The expression of PT11 under a constitutive promoter, can also help defining if cell specific variations in PT11 protein fusion levels is independently of transcriptional control.
-Lines 246-249: This conclusion is too strong and should be lowered down. See comments just above. You can't state that variations in PT11 reflect P transfer without actually showing this or providing other evidences to support it (e.g. for instance, does a mutated PT11 fusion variant impaired in P transfer still show cell specific variation levels? Ideally, PT11 localization could be done simultaneously with live sensors that enable the dynamic monitoring of phosphate levels in the cell (PMC4348774). Providing additional experimental proof would definitely strengthen these main findings of the paper.
-Lines 293-295: This conclusion is too restrictive. An alternative possibility is that 'only constructs that ensure sufficient PT11 expression levels in arbuscules retain PT11 function and enable successful AM colonisation'.
-Methodology: Number of independent experiments are not included in most cases. Revise along the document and include n number of individual samples, number of plants and number of independent experiments.

Minor comments
Line90 : describe for a non-specialist what is Tos17.
Line 254-255: construct also affect expression
Line 615: missing "for" after cambridge.

Version 1:

Reviewer comments:

Reviewer #1

(Remarks to the Author)
The authors have introduced all the changes I had recommended and I have no additional request.

Reviewer #2

(Remarks to the Author)
I would like to thank authors for addressing my initial comments fully and adequately. There is only one additional recommendation: although there are labels of the major membrane domains of arbuscule containing cells in Figure 1 (plasma membrane, peri-arbuscular membrane around the arbuscule trunk, thick and fine branches etc.) such labels must be applied for fluorescent images presented in Figure 2 to 7 to facilitate an interpretation of the results presented in the text of the manuscript.

Reviewer #3

(Remarks to the Author)
My specific comments and concerns have been adequately addressed by the authors. As such, the manuscript provides innovative, high-quality data that deserves to be published in Nature Communications. Only a few minor changes need to be made to the text prior to publication:
I am glad that the authors have carefully responded to my concern about the overstatement that PT11 transporter protein level directly reflected phosphate transport. They also provided relevant information on their unsuccessful attempts to address this question through the use of the FLIPPi biosensor Nevertheless, a few sentences in the manuscript remain slightly ambiguous on this point. I thus recommend that the authors carefully revise them:
-Lines 264-265: The sentence “This implies the differences in PT11-mRFP1 abundance are indeed reflective of nutrient transporter regulation” is misleading. The localization data indeed provide evidence that the higher PT11 signal intensity is specific to the PT11 transporter and not an overall response (as compared to scamp). While this data implies that levels of PT11 production or localization are upregulated, it does not provide evidence for or against nutrient transporter regulation. Please rephase.
-Lines 380-383: The statement here that “…but also the phosphate uptake capacity of those arbuscules, with a significant reduction in PT11 abundance (Fig. 6)” is too strong. No doubt that PT11 should be present for Pi transport. However, data in Fig 6 based on P11 localization cannot be used to infer phosphate uptake capacity. Please rephrase.
Other minor editing:
-Line 94: a point is missing just after “(Supplemental Fig. S1)”.
-Lines 108–112: The authors clarified on page 10 (point 4) that repeated daily imaging did not affect the progression of colonisation. This information should be included in the text or methodology section with a reference to the previous McGaley (2024) publication.

Open Access This Peer Review File is licensed under a Creative Commons Attribution 4.0 International License, which permits use, sharing, adaptation, distribution and reproduction in any medium or format, as long as you give appropriate credit to the original author(s) and the source, provide a link to the Creative Commons license, and indicate if changes were

made.
In cases where reviewers are anonymous, credit should be given to 'Anonymous Referee' and the source.
The images or other third party material in this Peer Review File are included in the article’s Creative Commons license, unless indicated otherwise in a credit line to the material. If material is not included in the article’s Creative Commons license and your intended use is not permitted by statutory regulation or exceeds the permitted use, you will need to obtain permission directly from the copyright holder.
To view a copy of this license, visit https://creativecommons.org/licenses/by/4.0/

Author responses to the reviewers' comments for submission **NCOMMS-25-64233**

Author response in blue

RESPONSE TO REVIEWERS

We wish to thank all three reviewers for their extensive and insightful comments on the original manuscript. Below please find the point-by-point responses to the reviewers' comments.

Reviewer #1 (Remarks to the Author):

The study by Mac Galey and colleagues presents an unprecedented insight into the cellular-level dynamics of arbuscular mycorrhizal symbiosis. By combining innovative non-invasive live imaging with elegant genetic approaches, the authors provide the most detailed temporal and spatial characterization to date of arbuscule development, collapse, and phosphate transporter dynamics. The discovery that arbuscules are not identical units of nutrient exchange, but display heterogeneity in lifespan, developmental trajectories, and PT11 abundance, is highly novel and of broad significance. These findings have profound implications for how we interpret AM colonization and evaluate symbiotic efficiency. Lastly, the use of different AM fungi with diverse colonization patterns and solid controls such as mutant phenotype complementation with the fluorescent protein fusion constructs give broad and sound support to the Author conclusions.

We thank the reviewer for their positive feedback on this manuscript, and for the following very helpful suggestions and comments.

I have noted a few minor points that may be improved:

1. While the reference list is extensive and covers most of the seminal literature, it would be worth double-checking whether all recent advances in single-cell or spatial transcriptomics in AM symbiosis have been cited, as these relate directly to the emerging concept of heterogeneity among arbusculated cells.

We thank the reviewer very much for this suggestion. We have now included references for recent single-cell and spatial transcriptomic studies, and reviews/outlooks for their application to AM symbiosis research (ln 373).

2. The manuscript is generally well written, but in some sections (especially Results), the density of detail occasionally hampers readability. The Authors might consider shortening or restructuring very long sentences to aid comprehension.

Thank you for this feedback. The text throughout the entire manuscript (but particularly the results) has been refined to aid readability and clarity.

3. the legend of figure 1A should include the indication of mid-grey color for the interface compartment

Thank you for noticing this omission. The grey colours have all now been properly referenced in the legend, and general labelling of Figure 1A has been made clearer.

4. Terms such as “collapse,” “degeneration,” and “senescence” are sometimes used interchangeably for arbuscule decline. A consistent terminology might improve clarity, unless different meanings are intentionally used.
Different meanings were not intentionally used, and as such we have now consistently used ‘collapse’ throughout the manuscript.

Reviewer #2 (Remarks to the Author):

In the manuscript titled “Symbiotic phosphate transporter dynamics in rice expose functional plasticity of the arbuscules,” McGaley et al. utilize stable transgenic lines of rice expressing fluorescent protein–tagged phosphate transporter PT11 to track and describe its presence on the periarbuscular membrane (PAM) throughout arbuscule development during mutualistic symbiotic interaction with arbuscular mycorrhizal (AM) fungi. The presented work is primarily descriptive (which is not a negative characterization) and provides remarkable new details through non-invasive imaging of fluorescently tagged reporters in colonized rice roots. Considering the inherent difficulties of such imaging, the effort and work are tremendous, and the primary author should be recognized and praised. The measurements of arbuscule lifespan are important and deserve great attention.

We thank the reviewer for their supportive feedback, and particular acknowledgement of the time and effort dedicated to the imaging-based experiments in this work.

1. However, the manuscript lacks adequate interpretation (and presentation) of the obtained data and suffers from exaggerated and embellished language, which diminishes the impact of the results. Moreover, more than half of the data (Figures 3 to 6) are based on the inferior fluorescent PT11-mRFP reporter, whereas the superior reporters PT11-GFP and PT11-mClover3 were used only as supplementary.

We thank the reviewer for their comments on the fluorescent reporters used in this work. We do not consider the PT11-mRFP1 reporter to be inferior on the grounds that firstly, the fluorescence distribution of PT11-mRFP1 mirrors that of PT11-eGFP, the only exception being the ability of mRFP1 to continue fluorescing once sent to the vacuole due to its lower pKa (which we highlight in ln 166 and Fig. S6). To ensure this is completely clear, we have updated the images in Fig. 3Ai-v, as well as included an additional supplementary figure, Supplemental Fig. S7A. This figure shows the sharp PT11-mRFP1 signal around arbuscule branches and well-defined twin intensity peaks in the fluorescence intensity plot of a transect through an arbuscule branch. Secondly, the construct can complement the *pt11* mutant phenotype, proving it is functional (Fig. S5B). And thirdly, the PT11-mRFP1 distribution contrasts strongly with the distribution of ‘free’ mRFP1 in arbusculated cells (Fig. S7A), showing that there is no fluorophore cleavage or protein mislocalisation.

PT11-mRFP1 was used in the majority of the study due to the spectral separation between mRFP1 and eGFP. It could therefore be combined with eGFP-SCAMP to provide an arbuscule marker and internal fluorescence control.

Regarding the reviewer's suggestion of 'exaggerated and embellished language', no examples are specified and we are therefore uncertain about which 'language' they refer to. The concerns about language highlighted in reviewer's comment #2 are addressed below.

2. The title and the summary paragraph of the introduction (lines 67–80) exemplify exaggerated language and interpretations that are not supported by the data. There is no evidence supporting the claim of "functional plasticity of the arbuscules" (title) or the statement "…uncovered a cellular-level of nutrient regulation of symbiotic phosphate uptake critical to symbiotic success. The observed capacity for symbiotic nutrient exchange to be fine-tuned at the arbuscule level" (lines 74–76). The presented work only demonstrates the fluorescence of PT11-FP at different stages of development and different conditions and does not show actual phosphate transport at any stage of arbuscule development to justify such statements.

We agree with the reviewer that it would be ideal to monitor both phosphate transporter protein AND phosphate transport activity at the single-cell level. The only existing approach we are aware of is using phosphate biosensors. We tried the previously-published FLIPPi biosensor construct (Zhang et al., 2022) in rice, but unlike the original study in Brachypodium, several factors that made phosphate monitoring via FLIPPi impossible: one of the FRET partners was very weakly fluorescent, there was extremely high variation in FRET, and there was no responsiveness of FRET ratios to phosphate treatment or mycorrhizal colonisation. We have included example data displaying this below (Reviewer Figure 1-3).

[FIGURE REDACTED]

Reviewer Figure 1. Representative images of root cortical cells in rice expressing FLIPPi biosensor from Zhang et al., (2022). Images show weak eCFP expression, requiring minimum 30% laser power to gain weak signal (b). At this laser power, much of the signal is background autofluorescence.

[FIGURE REDACTED]

Reviewer Figure 2. High variability in FLIPPi biosensor FRET ratio between rice roots (single data points = average of entire root), plants and plant age, with no significant effect of phosphorus fertilisation. In older plants, roots are more autofluorescent, leading to altered FRET ratio measurements. LP, (low phosphate, 25uM) or HP (high phosphate, 250uM) fertilizer was applied twice per week.

[FIGURE REDACTED]

Reviewer Figure 3. Comparison of FRET ratios measured from FLIPPi biosensor in rice (a) and published Brachypodium data ((b), from Zhang et al., (2022)) upon cell colonisation by AM fungi. A = arbusculated cell, N = non-colonized cortical cell. No change was detected using FLIPPi biosensor in rice (data from two representative plants shown).

Due to the technical impossibility of monitoring phosphate transport, we are therefore clear throughout the manuscript that we are looking at a 'proxy' (ln 20) or 'indicator' (ln 68) for phosphate transport, or phosphate transport 'capacity' (lns 23, 76, 382). We feel it is correct to use phosphate transporter abundance as an indicator of phosphate transport capacity, because the transporter protein PT11 is the enabler of symbiotic phosphate transport (Yang et al., 2012). Without PT11, phosphate transport cannot take place (zero capacity for phosphate transport). In contrast, when PT11 is abundant, the 'capacity' for phosphate transport is higher. Even it if there are further levels of regulation of actual nutrient transport, PT11 must first be present before phosphate transport can occur.

As phosphate transport is one of the key 'functions' of the arbusculated cell, and its capacity for phosphate transport changes in response to nutrient availability (ability to change in response to environmental factors is the definition of 'plasticity'), we believe the phrase "functional plasticity of the arbuscules" accurately conveys the concept in question.

For additional clarity, we have changed wording from 'transport' to 'transporter' or 'importer' where relevant (e.g. lns 76, 265, 343, 391). This addresses the reviewers specific concern about the phrase "…uncovered a cellular-level of nutrient regulation of symbiotic phosphate uptake critical to symbiotic success" as we now specify phosphate 'importer' (ln 76).

3. Figure 1
It is emphasized that SCAMP-GFP accumulates at branch tips at stages ii and iii (Fig. 1B). Such accumulation is not visible. Why is it important that SCAMP-GFP accumulates at fine branches, given that it is a marker of the plasma membrane and PAM?

The more intense eGFP signal at branch tips is indicated by the 'whiter' pixels. This is striking when compared to the eGFP intensity around coarse branches that are barely visible, and the trunk which is very faint.

It is important to account for tip-accumulation of eGFP-SCAMP when interpreting the following micrographs of the *eGFP-SCAMP ; PT11-mRFP1* co-expression line (Fig. 3), where eGFP-SCAMP is used as a reference. For example, if eGFP-SCAMP was evenly distributed over the entire PAM, then one might interpret Figure 3ciii as showing absence of PT11-mRFP1 from the branch tips, when in fact, eGFP-SCAMP is just more abundant there. We have added the individual channels for clarity in Supplemental Fig. S8, along with the fluorescence intensity measurements.

4. Supplemental Fig. S2A–H is beautiful and informative and should be presented as a main figure along with the data in Fig. 1C.

The timelapse Movie 1 was intended to demonstrate the diversity in developmental trajectories, and these are supplementary snapshots to guide the readers eye through the developmental stages. We therefore prefer to direct readers to the actual timelapse Movie 1, but retain Fig. S2 in the supplemental as a guide for those less familiar with the arbuscule developmental stages.

5. It is unclear why the term “coarse” arbuscule branches is introduced instead of the commonly used “thick” branches. This change should be clarified and explained.

The term ‘coarse’ was used in line with some of the earliest detailed descriptions of arbuscule structure (e.g. Lackie et al. (1987), *Symbiosis*) and more recent reviews of arbuscule cellular biology (e.g. Gutjahr & Parniske (2013) *Annu. Rev. Cell Dev. Biol.* Journal). This word choice can help avoid confusion when comparing morphologically diverse arbuscules. ‘Thick’ and ‘thin’ can be used as comparative size descriptors, and ‘coarse’ and ‘fine’ can refer to the developmental position in the arbuscule.

6. Figure 2
SCAMP-GFP is a marker of the plasma membrane and PAM. However, it is very difficult to see well-defined labeling of these two membranes in the presented images. One must assume it is on the membranes. While it is understandable that obtaining high-quality images is challenging, the images presented should be clear and representative. Such clear presentation is especially important for newcomers in the field who are learning about the cell biology of AM symbiosis.

We disagree with the reviewer and believe these images are of comparable quality to our initial SCAMP-eGFP characterisation in Fig. 1, the labelling of which should assist any newcomers to the field interpret the symbiotic structures that are featured.

7. Figure 3
This figure introduces a new fluorescent reporter of PT11 as a fusion to mRFP and describes the creation of a rice stable transgenic line co-expressing PT11-mRFP and GFP-SCAMP for co-localization studies. This transgenic line was used to assess PT11-mRFP and GFP-SCAMP location at different stages of arbuscule development. However, PT11-mRFP appears to be a highly inferior reporter, as it is impossible to see it on the PAM; the signal is visible primarily as cytoplasmic and vacuolar haze (Fig. 3B). It is puzzling that the manuscript states PT11-mRFP locates on the PAM similarly to the PT11-GFP reporter (Fig. 3A). There is no similarity in the fluorescent pattern, and it is misleading to claim this (lines 160–161).

Please see response to the reviewer's first comment. We trust that the new, better resolved Supplemental Figure S7 removes any doubt about the localisation (especially when compared to true nucleocytoplasmic 'free' mRFP1).

8. Figures 3C and S6 present simultaneous localization of PT11-mRFP and GFP-SCAMP or PT11-mRFP and PT11-GFP, respectively. Separate images showing only PT11-mRFP fluorescence must be presented in addition to the overlay images in both figures.

This is a very useful suggestion, thank you. This has accordingly been added to Supplemental Fig. S8.

9. Lines 176–177: "…but all PT11 was removed from the PAM at collapsed arbuscules." How was the removal of PT11 monitored? This should be clarified.

This is a good point- we have captured removal using the timelapse approach (Movie 2), however this paragraph refers to the static imaging so we have rephrased to "…but PT11 was absent from the PAM at collapsed arbuscules…" (ln 187).

10. Figure 4
The PT11-mRFP reporter was used to monitor its location at different stages of arbuscule development from different species of AM fungi. Similar to the previous figure, it is difficult to see PT11-mRFP on the PAM; the signal appears mainly as fluorescent haze. The statement "PT11 was still visible only in cells hosting young and mature arbuscules…" (lines 202–203) is insufficient and incorrect, as fluorescence from PT11-mRFP (magenta) is shown. PT11 should be on the PAM, which is not evident in the presented images.

This experiment has been repeated using the *PT11-mRFP1 ; eGFP-SCAMP* co-expression line to show the fungal structures more clearly. This is presented in the updated Fig. 4. We have stated that the mRFP1 remains fluorescent in the vacuole in ln 167, so this should be expected at the Collapsed arbuscule stage.

11. Images in Fig. 4E are particularly inadequate, as it is almost impossible to discern anything in any light channel.

This has now been repeated with eGFP-SCAMP ; PT11-mRFP1 co-expression line with four different fungal species to highlight the symbiotic structures to which PT11-mRFP1 does not localise (intracellular hyphae, trunks, collapsed arbuscules). This is presented in updated Fig. 4, with Ai-ii, Bi-ii, Ci-ii and Di-ii showing the intracellular hyphae.

12. Figure 5
The measurements presented in Fig. 5 are based on the use of the "poor" PT11-mRFP reporter (see above). In the corresponding section of the manuscript (lines 210–223), it is concluded that PT11 shows high variability in abundance on the PAM. It is unclear how such a conclusion can be made, given that no clear evidence has been presented showing PT11-mRFP localization on the PAM.

Please see our response to comment #1 about the PT11-mRFP1 reporter, specifically the inclusion of better resolved images in Fig. 3 and the additional Supplemental Fig. S7A. Further, this data was

taken from transects drawn around branch tips, so none of the vacuolar mRFP1 is included. We therefore disagree with this reviewer comment.

13. Figure 6
Similar comments apply to the data in Fig. 6. Higher PT11-mRFP intensities are detected under lower phosphate conditions (line 239). Were these higher intensities detected on the PAM? This is unclear from the images.

This dataset was analysed twice, once taking measurements from individual fine branches of the arbuscules, and once taking the mean fluorescence of the total arbuscule area. The results were the same, and also mirrored with the PT11-mClover3 reporter (Fig. 7B). We are therefore confident that the different mRFP1 intensities represent different PT11-mRFP1 abundance in the PAM.

14. In the corresponding section of the manuscript (lines 226–249), the word "confirming" is used extensively to describe the data. This could indicate confirmational bias rather than critical interpretation of the results.

Thank you for this useful observation. The section of the manuscript has been re-worded so that 'confirm' is correctly used. As such, Ln 259 now reads "…proving that PT11 abundance on the PAM is responsive to plant phosphate demand" and Ln 267 now reads "..verifying that the properties of the fluorescent tag were not responsible for the observed fluorescence intensity differences under different nutrient regimes…".

15. Figure 7
This is the best data in the entire manuscript. The fluorescent images of PT11-mClover3 are beautiful and high-quality, with clear localization of the reporter on the PAM. It is puzzling why this reporter was not used in all analyses presented in the previous figures.

We thank the reviewer for these comments. A very large body of work and characterisation had been completed before the *PT11-mClover3* line was ready, due to the timeframe required to generate stable transformants in rice. For proper characterisation of PT11 dynamics, it was also necessary to compare against the known PAM marker eGFP-SCAMP, which could not be completed with the PT11-mClover3 line due to spectral similarities.

16. Additionally, the figure legend is highly informative and detailed, in contrast to the legends for earlier figures. In general, Fig. 7 should be the first figure of the manuscript.

Thank you for this feedback. Figure legends have been now improved throughout the entire manuscript.

We disagree that Fig. 7 should be the first figure of the manuscript as its interpretation requires readers to understand arbuscule dynamics and developmental stages (Fig. 1), PT11 dynamics and localisation (Fig. 2-4), the concept of variable PT11 abundance at the arbuscule (Fig. 5-6), the supplemental data relating to the *pt11* mutant phenotype and complementation by PT11 reporter constructs (Fig. S4-5), and SCAMP regulation by nutrient levels (Fig. S13).

17. The data in Fig. 7B are robust and informative, in contrast to the data in Fig. 6B, which show only

a minuscule difference in fluorescence intensities. Fig. 7 uses “mean photon count,” whereas Fig. 6B uses “mean gray value.” Why were different metrics used? This should be explained and added to the figure legend.

The initial work was performed on a Leica SP8 with detectors in ‘digital’ mode, giving gray value data. After the research group moved department, a Leica Stellaris FALCON/FLIM 8 with photon counting detectors was used. This information has now been added to the methods section (ln 609).

18. Line 245-249. It seems that this sentence is not complete.

This has been changed to “…verifying that properties of the fluorescent protein tag were not responsible for the observed fluorescence intensity differences under different nutrient regimes.” (Ln 267)

19. Line 293-295. What this sentence mean?

The constructs that maintained the nutrient regulation of PT11 abundance at the arbuscule (*pSTR1:PT11-mClover3* and *pPT11:mClover3*) could complement the *pt11* mutant phenotype, suggesting they were functional. This is despite *pSTR1:PT11-mClover* altering PT11 localisation.

Reviewer #3 (Remarks to the Author):

Manuscript of McGaley et al.

General comments
This manuscript focuses on the study of important root symbiosis between plants and arbuscular mycorrhizal (AM) fungi, forming specialized hyphal structures known as arbuscules, for nutrient transfer (particularly phosphate) to the host plant. In this work, the authors employed live cell imaging methods along with validated fluorescent markers (the membrane marker SCAMP and PT11 phosphate transporter, as a proxi of P transport) to investigate the developmental trajectory of arbuscules and PT11 transcription/protein localization at cellular resolution. This research provides novel insights into (i) the dynamic phases of arbuscule growth and collapse within individual rice cells; (ii) the localization of PT11 at specific stages of arbuscule development across different AM fungal interactions; and (iii) the cell-specific regulation of PT11 abundance in individual arbuscules, which the authors demonstrate to be under nutrient (P) regulation.
The results are based on appropriate methodologies and tools, producing high-quality data that are generally well supported by robust analysis. Overall, the findings are compelling and contribute to our understanding of the cellular steps underlying arbuscule development and collapse and the contribution of P11 contribution to this process. Most of the analyses rely on the use of SCAMP and PT11, which have been previously validated as useful markers for studying arbuscule development in rice. The novelty here lies in their use in fine cellular resolution studies, including original colocalization experiments, producing new descriptive and quantitative data on developmental trajectories of individual arbuscules, in addition to providing cellular information on PT11 promoter activity and protein localization in branched arbuscule stage. The conclusions drawn in these sections are sound and well supported by the data, although improvements to figure descriptions and

labelling—particularly to assist non-specialist readers—are required to enhance clarity (as outlined below).
The manuscript also presents robust data on the cell-autonomous regulation of PTR11 abundance in individual cells and clearly demonstrates that this abundance is influenced by nutrient (P) conditions. This provides evidence for a cellular layer of nutrient regulation, which is very interesting. However, I have a concern about the overstatement that this change in abundance directly reflects the regulation of nutrient transport, which may be the case, but this is not what the data actually show. Such a conclusion requires additional supporting evidence (outlined below).

We thank the reviewer for their summary of this work, and for the very detailed and helpful comments. We believe the manuscript is a lot clearer and stronger thanks to the changes suggested.

Specific comments:
1. Line 67. PT11 is used as a proxi of P transport, however, the rationale behind using PT11 rather than another P transporter should be explained. To what extent can general conclusions about P transport be drawn only on the basis of P11? Would it not have been relevant to at least compare the dynamics of PT11 with those of another P transporter?

Thank you for this comment. We focussed on PT11 based on the study of Yang et al., (2012), in which PT11 is shown to be the single essential, symbiosis-specific member of the PHT1-family phosphate importer in rice responsible for 70% of total phosphate uptake during AM symbiosis.

2. Line 87: Many experiments in the manuscript rely on the use of SCAMP and PT11, used in previous studies, yet background information on these markers is not well developed in the text. Please provide this background information to better highlight what is new here relative to these previous studies.

Thank you for this feedback. For the PT11-eGFP marker, we reference Kobae & Hata (2010) for their study showing symbiotic phosphate transporter localises to the arbuscule fine branches (introduction, lns 53-57), and have added the citation of Yang et al 2012 in our initial introduction to the PT11 transporter (introduction, ln 68). We have added extra background information into the results to acknowledge previous work conducted with the PT11-eGFP construct by Kobae & Hata (2010) (Ln 140-143), and discuss these findings in our discussion (lns 346-351).

For the eGFP-SCAMP marker, we introduce its use as a PAM marker at the start of our results section, citing Kobae and Fujiwara (2014), and have now added the additional reference of Kobae et al., (2016), highlighting the “characteristic distributions (of SCAMP) at each arbuscule developmental stage” (Lns 87-91). We then refer to previous insights gained and what new has been achieved in this work in the discussion Lns 322-323 and lns 325-328.

3. Fig. 1: The data description is not always accessible to non-specialists. It is not always obvious what is being described in the fluorescence images. Bright field images should be included to visualise cell contours and arbuscules. Label the diverse structures in the images (e.g. coarse and fine branches, trunk, etc.). Despite the arrowheads, transvacuolar strands are not clearly visible either. 'Cb' refers to both coarse branches and collapsing branches (please modify this). Clarify the labelling in the other figures as well.

Thank you for this feedback. We have now improved the labelling of Figure 1 and hope that this can act as a reference for non-specialists, to help them interpret the full manuscript. A different section

of the z-stack for Fig. 3Bv has been selected to better show the transvacuolar strands. We have also improved the selection of micrographs shown throughout the manuscript, so it should be clearer for non-specialists (e.g. Figure 3, Figure 4, Supplemental Figure S7, Supplemental Figure S8). Unfortunately the confocals used are not equipped with DIC, and the arbuscules are not visible under normal brightfield illumination.

4. Lines 108-112: 15% of arbuscules collapse quickly. How consistent is this number across different experiments? Could the collapse be triggered by the experimental conditions, which are based on repeated observations of samples using confocal microscopy? A control experiment without repeated observations could help determine the biological significance of this variation.

We agree this important control, and it was indeed performed as part of the AMSlide publication (McGaley et al., 2024) which showed that repeated daily imaging did not to affect the progression of colonisation. These timelapses have been repeated at various intervals over 5 times, with similar variation in arbuscule development and lifespan observed.

5. Line 217: The inter-root-level variations in PT11 abundance should be better explained. Providing information on root features (size and diameter), developmental stage (growing region or older part of roots) or root type (main vs adventitious vs lateral roots?) from where the arbuscule data was extracted could help understanding it.

Thank you for this suggestion. We have now included information about which root type was imaged (Ln 235). As these roots were of the same type, developmental stage, and size, we have no evidence from this dataset that the inter-root variations in PT11 abundance are reflective of underlying tissue differences.

6. Line 225: From the data shown in Fig 7, it is concluded that the abundance in PT11 protein fusion levels is variable in different LP vs HP nutrient conditions. This is indeed a coherent conclusion, reflecting what the data really shows. However, in this section and throughout the manuscript, there is an overstatement of this conclusion, with 'functional' inferences about P transport being drawn from this data. It is important to remove this ambigous statetement from the text. Additional experimental proof is also needed to understand the significance of these cell-specific variations in PT11 levels. First, it is important to verify if variations in PT11 levels seen in LP/HP conditions is not simply reflecting fine-transcriptional regulation of PT11 promoter that is driving fusion expression. Using the PT11 transcriptional fusion (Fig 2) or in co-dynamic studies with the P11 protein fusion in LN and HN conditions, can help determining that. The expression of PT11 under a constitutive promoter, can also help defining if cell specific variations in PT11 protein fusion levels is independently of transcriptional control.

Thank you for this feedback. We have addressed the concern about inferring phosphate transport from transporter protein presence in our response Reviewer #2's comment #2 above.

The fact that NLS-TurboRFP stably accumulates in the nucleus hampered efforts to monitor PT11 promoter activity in plants exposed to LP vs HP. However, based on the promoter swap lines, it seems likely that both transcriptional and translational regulation is involved in fine-tuning final PT11 abundance in the PAM. As discussed in Ln 413, the *pSCAMP:PT11-mClover3* line showed no response (protein abundance) to LP vs HP. This contrasts with the *pSCAMP:eGFP-SCAMP* line, despite sharing a promoter, and *pPT11:PT11-mClover3,* despite sharing protein sequence.

7. Lines 246-249: This conclusion is too strong and should be lowered down. See comments just above. You can't state that variations in PT11 reflect P transfer without actually showing this or providing other evidences to support it (e.g. for instance, does a mutated PT11 fusion variant impaired in P transfer still show cell specific variation levels? Ideally, PT11 localization could be done simultaneously with live sensors that enable the dynamic monitoring of phosphate levels in the cell (PMC4348774). Providing additional experimental proof would definitely strengthen these main findings of the paper.

Thank you for these suggestions. We in fact did try the FLIPPi biosensor in rice, however it has not been effective (please see the data and response to Reviewer 2 comment #2). Given this technical impossibility of directly monitoring phosphate transport, using phosphate transporter presence and abundance as an indicator of phosphate transport capacity is the best existing option. Please see our response to Reviewer #2's comment #2 above for further details of how we have clarified that we are reporting an 'indicator' phosphate transport, not directly monitoring phosphate transport.

8. Lines 293-295: This conclusion is too restrictive. An alternative possibility is that 'only constructs that ensure sufficient PT11 expression levels in arbuscules retain PT11 function and enable successful AM colonisation'.

This is an important point, thank you for raising it. The fact that there is a large overlap in PT11 abundance at the arbuscules under LP between pSCAMP:PT11-mClover3 and the other two constructs, but a very binary difference in arbuscule phenotype (Fig. 7B-D), implies that PT11 expression level alone is not the cause of failed complementation by *pSCAMP;PT11-mClover3.*

9. Methodology: Number of independent experiments are not included in most cases. Revise along the document and include n number of individual samples, number of plants and number of independent experiments.

Replicate information is now included, thank you for pointing this out.

Minor comments
10. Line90 : describe for a non-specialist what is Tos17.

Thank you for highlighting this omission. We have now included extra information about Tos17 in lns 91-92 and lns 447-449.

11. Line 254-255: construct also affect expression

We have addressed this comment above in response to Reviewer #3 comment #8.

12. Line 615: missing "for" after cambridge.

Thank you for noticing this, it has now been corrected.

REVIEWERS' COMMENTS (revised manuscript NCOMMS-25-64233A)

Author responses to Reviewers in blue.

Reviewer #1 (Remarks to the Author):

The authors have introduced all the changes I had recommended and I have no additional request.

Reviewer #2 (Remarks to the Author):

I would like to thank authors for addressing my initial comments fully and adequately. There is only one additional recommendation: although there are labels of the major membrane domains of arbuscule containing cells in Figure 1 (plasma membrane, peri-arbuscular membrane around the arbuscule trunk, thick and fine branches etc.) such labels must be applied for fluorescent images presented in Figure 2 to 7 to facilitate an interpretation of the results presented in the text of the manuscript.

We have improved the labelling in Figure 1A and 1B such that this is now sufficiently detailed to guide interpretation of the rest of the micrographs in the manuscript, which all feature the same subcellular structures. Labelling of all domains in all micrographs would obscure the actual patterns and intensities being displayed. The addition of labelling to Figure 1 was also suggested by the other two Reviewers, and they are now content with the changes made.

Reviewer #3 (Remarks to the Author):

My specific comments and concerns have been adequately addressed by the authors. As such, the manuscript provides innovative, high-quality data that deserves to be published in Nature Communications. Only a few minor changes need to be made to the text prior to publication:
I am glad that the authors have carefully responded to my concern about the overstatement that PT11 transporter protein level directly reflected phosphate transport. They also provided relevant information on their unsuccessful attempts to address this question through the use of the FLIPPi biosensor Nevertheless, a few sentences in the manuscript remain slightly ambiguous on this point. I thus recommend that the authors carefully revise them:

-Lines 264-265: The sentence “This implies the differences in PT11-mRFP1 abundance are indeed reflective of nutrient transporter regulation” is misleading. The localization data indeed provide evidence that the higher PT11 signal intensity is specific to the PT11 transporter and not an overall response (as compared to scamp). While this data implies that levels of PT11 production or localization are upregulated, it does not provide evidence for or against nutrient transporter regulation. Please rephase.

Thank you for this suggestion. We have now removed ambiguity by changing ‘nutrient transporter’ to ‘symbiotic phosphate transporter’, as it is correct that we are only discussing PT11 here, not all nutrient transporters (ln 265-267)

-Lines 380-383: The statement here that "…but also the phosphate uptake capacity of those arbuscules, with a significant reduction in PT11 abundance (Fig. 6)" is too strong. No doubt that PT11 should be present for Pi transport. However, data in Fig 6 based on P11 localization cannot be used to infer phosphate uptake capacity. Please rephrase.

Thank you for this comment. We have rephrased this sentence to make it clearer that our observation of reduced PT11 abundance under HP fertilisation is only a 'potential indicator' of reduced phosphate uptake capacity of the arbuscules. (ln 381-384).

Other minor editing:
-Line 94: a point is missing just after "(Supplemental Fig. S1)".

Thank you for noticing this- it is now corrected.

-Lines 108–112: The authors clarified on page 10 (point 4) that repeated daily imaging did not affect the progression of colonisation. This information should be included in the text or methodology section with a reference to the previous McGaley (2024) publication.

This is a good suggestion and has now been added to the methods section (ln 575-576)
